# Classification of Mouse Retinal Bipolar Cells: Type-Specific Connectivity with Special Reference to Rod-Driven AII Amacrine Pathways

**DOI:** 10.3389/fnana.2017.00092

**Published:** 2017-10-24

**Authors:** Yoshihiko Tsukamoto, Naoko Omi

**Affiliations:** ^1^Studio EM-Retina, Nishinomiya, Japan; ^2^Department of Biology, Hyogo College of Medicine, Nishinomiya, Japan

**Keywords:** mouse retina, bipolar cell, ribbon synapse, amacrine cell, retinal ganglion cell, gap junction, serial section transmission electron microscopy (SSTEM), microcircuital connectome

## Abstract

We confirmed the classification of 15 morphological types of mouse bipolar cells by serial section transmission electron microscopy and characterized each type by identifying chemical synapses and gap junctions at axon terminals. Although whether the previous type 5 cells consist of two or three types was uncertain, they are here clustered into three types based on the vertical distribution of axonal ribbons. Next, while two groups of rod bipolar (RB) cells, RB1, and RB2, were previously proposed, we clarify that a half of RB1 cells have the intermediate characteristics, suggesting that these two groups comprise a single RB type. After validation of bipolar cell types, we examined their relationship with amacrine cells then particularly with AII amacrine cells. We found a strong correlation between the number of amacrine cell synaptic contacts and the number of bipolar cell axonal ribbons. Formation of bipolar cell output at each ribbon synapse may be effectively regulated by a few nearby inhibitory inputs of amacrine cells which are chosen from among many amacrine cell types. We also found that almost all types of ON cone bipolar cells frequently have a minor group of midway ribbons along the axon passing through the OFF sublamina as well as a major group of terminal ribbons in the ON sublamina. AII amacrine cells are connected to five of six OFF bipolar cell types via conventional chemical synapses and seven of eight ON (cone) bipolar cell types via electrical synapses (gap junctions). However, the number of synapses is dependent on bipolar cell types. Type 2 cells have 69% of the total number of OFF bipolar chemical synaptic contacts with AII amacrine cells and type 6 cells have 46% of the total area of ON bipolar gap junctions with AII amacrine cells. Both type 2 and 6 cells gain the greatest access to AII amacrine cell signals also share those signals with other types of bipolar cells via networked gap junctions. These findings imply that the most sensitive scotopic signal may be conveyed to the center by ganglion cells that have the most numerous synapses with type 2 and 6 cells.

## Introduction

Recently, transcriptomics experiments defined 15 types of bipolar cells in the mouse retina (Shekhar et al., 2016). These molecular genetic data are consistent with the reported morphological data. Ghosh et al. (2004) first described 10 cell types (T1–T9 and RB), Mataruga et al. (2007) further divided the type 3 cells into T3a and T3b, then Wässle et al. (2009) presented a systematic survey of cone contacts, mosaics, and territories of the 11 identified types of bipolar cells. These analyses combined several methods, including dye injection, immunostaining, and experimental manipulation of transgenic mice. Similar results were obtained by Badea and Nathans (2004) using a genetic reporter and by Pignatelli and Strettoi (2004) using a gene gun. An increase in the sample size of bipolar cells reconstructed by serial block-face scanning electron microscopy (SBSEM) led to the identification of a novel X type cell (Helmstaedter et al., 2013) and the division of type 5 cells into inner (5i), outer (5o), and thick (5t) types (Greene et al., 2016). Lastly, Della Santina et al. (2016) revealed a peculiar type of bipolar cell which has no dendrites using dye injection and further described the distribution of axonal ribbons by SBSEM.

There are currently two different procedures of electron microscopy for microcircuital connectome, SBSEM (Helmstaedter et al., 2011), and SSTEM: serial section transmission electron microscopy (Anderson et al., 2011). Because membrane-to-membrane apposition without any synaptic contact may occur from place to place in neuropiles, direct visualization of chemical synapses and gap junctions is required for determining all synaptic connections (Anderson et al., 2011). In this respect, as compared to SBSEM, SSTEM is uniquely capable of identifying gap junctions with the aid of section tilting for observation angle adjustment. Furthermore, to determine whole retinal microcircuits, we need complete taxonomy of retinal neurons. Classification of all bipolar cells is one of the important steps to analyze the core part of retinal microcircuits.

**Figure 1:**
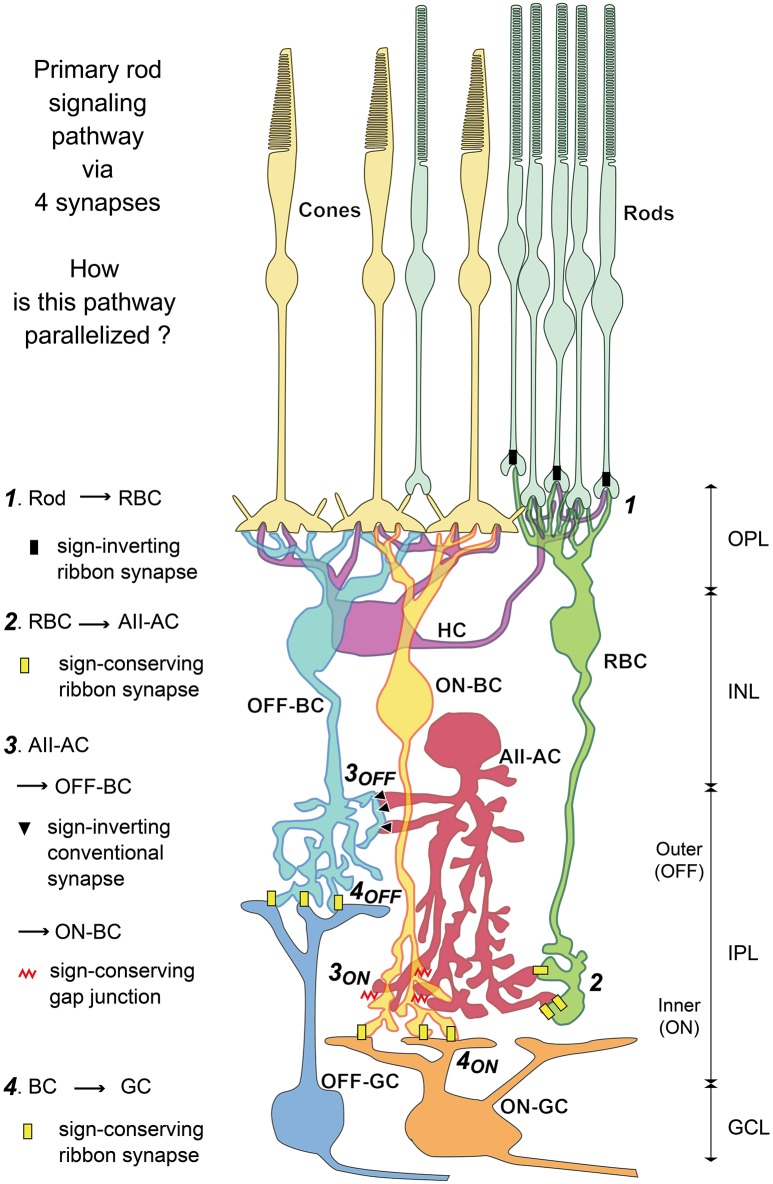
Diagram of primary rod-driven signal pathways via four synapses: rods → rod bipolar cell (RBC) → AII amacrine cell (AII-AC) → OFF or ON (cone) bipolar cell (OFF-BC, ON-BC) → OFF or ON ganglion cell (OFF-GC, ON-GC) as recognized from previous studies. The main aim of this study was to clarify the parallelism of this diagram based on the validation of all bipolar cell types. We evaluate the type-specific connections of bipolar cells with AII amacrine cells via conventional synapses and gap junctions. Furthermore, we observe type-specific bipolar-bipolar gap junctions (not shown in this diagram) extensively. OPL, outer plexiform layer; INL, inner nuclear layer; IPL, inner nuclear layer; GCL, ganglion cell layer.

The AII amacrine cell has been regarded as an important model neuron for retinal connectome (Lauritzen et al., 2013; Marc et al., 2014). The AII cell connects with almost all bipolar cell types for collecting scotopic signals from rod bipolar (RB) cells and distributing them to ON and OFF cone bipolar cells via gap junctions and inhibitory synapses respectively (Figure 1). It also mediates photopic inhibitory crossover signals between ON and OFF layers. In addition, it connects with many ganglion and other amacrine cell types. Marc et al. (2014) examined the synaptic architecture of rabbit retinal AII amacrine cell using SSTEM with molecular marker enhancement. They clarified AII amacrine connections with 28 different cell types in total. However, they did not describe the numbers of chemical and electrical synaptic contacts with “type-identified” bipolar cells per AII amacrine cell. To do such type-specific counts, we need the identification of the types of all bipolar cell processes contacting the AII amacrine cell of interest, after all bipolar cell types are validated.

In the first half of the results section, we validate the classification of bipolar cell types based on the distribution of synaptic ribbons in axon terminals and morphological parameters, including stratification level, arbor area, and arbor thickness. Wässle et al. (2009) suggested that type 5 cells might be divided into two groups mainly based on the coverage factor. By contrast, Greene et al. (2016) insisted that type 5 cells should be divided into three groups based on the detailed morphology of the axon terminal arbors and the coverage factor. However, morphological differences among the 5i, 5o, and 5t types are very subtle. Therefore, further characterization of type 5 cells at the level of synaptic contacts will contribute to eliminating ambiguity. Pang et al. (2004, 2010) suggested two distinct groups of RB cells: one (RB1) with a deeper axon terminal and less chloride channels than the other (RB2). We classified RB cells by inspecting the morphological counterparts of their physiological specifications. In particular, we sought to determine whether RB1 cells make membrane-to-membrane contacts with ganglion cell somas, as depicted by Cajal (1893) at the light microscopic level. Once determined, we investigated the possible existence of synaptic structures. Using cluster analysis, we assessed whether these two groups of RB cells are truly different or two variants of a single cell type.

In the second half of the results section, we initially assess the general relationship of bipolar cells with amacrine cells and next the connection strength of bipolar cells with AII amacrine cells (Figure 1). Amacrine cell-mediated inhibitory masking of bipolar cell axonal output was recently shown to work in association with three coordinated parallel pathways of rod signals (Pan et al., 2016). Masking mechanisms ensure the passage of appropriate signals from axon terminals of all types of bipolar cells. Therefore, first, we compared the density of contacts between input amacrine synapses and output ribbon synapses along every type bipolar cell axon. During this examination, we pay attention to ectopically midway ribbons along the ON bipolar cell axons passing through the OFF sublamina. Second, to clarify how the rod signal is electrically coupled to different types of bipolar cells, we investigated gap junctions between AII amacrine and ON cone bipolar cells, among ON cone bipolar cells, and among OFF cone bipolar cells. Previously, bipolar cell type-specific gap junctions were almost unknown. Third, by discriminating between direct and indirect (via OFF cone bipolar cells) OFF rod signaling pathways, we examined the allocation of chemical synapses between AII amacrine and ganglion cells. Finally, we evaluated the relative contributions of bipolar cell types to relaying ON and OFF rod signals in parallel from AII amacrine cells to ganglion cells.

It is highly likely that all types of mouse bipolar cells are now available for formulating comprehensive pathways. Therefore, we have attempted to characterize the system architecture of neuronal circuits in the mouse retina, especially of the primary rod signaling pathways. Our previous report (Tsukamoto and Omi, 2013) concentrated on the former half of the circuit: rod cells → RB cells → AII amacrine cells. This report is concentrated on the latter half: AII amacrine cells → OFF (chemical synapses) and ON (gap junctions) bipolar cells → ganglion cells, as well as some classification issues.

## Materials and methods

### Tissue preparation and electron microscopy

For 3D reconstruction of retinal neurons in this study, we used the same series of electron micrographs of the central retina of the mouse (C57BL/6J, female, 20 g, 9 weeks old; provided by Japan SLC, Inc., Shizuoka, Japan) as described in our previous studies (Tsukamoto et al., 2001; Tsukamoto and Omi, 2013). In brief, the mouse was deeply anesthetized with sodium pentobarbital (45 mg/kg i.p.) and perfused with a fixative containing 2% paraformaldehyde, 2.5% glutaraldehyde, and 1% acrolein in phosphate buffer (0.1 M, pH 7.4). The right eyeball was enucleated, and the posterior pole of the retina was immersed in the same fixative, with 1% tannic acid replacing the acrolein. The tissue was postfixed with 1% osmium tetroxide for 2 h, stained with 3% uranyl acetate in 80% methanol, dehydrated with ethanol, and embedded in araldite resin. All animal experimental procedures were approved by the Hyogo College of Medicine Committee on Animal Research and were performed in accordance with the Act on Welfare and Management of Animals issued by the government of Japan.

A series of 366 radial sections were cut at a thickness of 90 nm. Sections were mounted on formvar-covered single-slot grids, stained with uranyl acetate and lead citrate, and photographed at 3,000X using JEM1200EX and JEM1220 electron microscopes (JEOL Ltd., Tokyo, Japan) at the Joint-Use Research Facilities, Hyogo College of Medicine. After 4-fold enlargement on print paper, we obtained images for analysis at a final magnification of 12,000X. The contour lines of neurons and the sites of individual chemical and electrical synapses were identified by human pattern recognition and drawn on consecutive transparent sheets using color pens. After digitization, the 3D images were reconstructed on a personal computer using TRI/3D-SRF-R graphic software (Ratoc Systems International, Inc., Tokyo, Japan) for Windows 8. Selected areas of chemical synapses and gap junctions were rephotographed at 40,000X with various tilts. For graphical representations, we used Photoshop and Illustrator in Adobe CS6 (Adobe Systems Inc., San Jose, CA, USA).

### Multivariate analysis of clustering with morphological measurements

Classification of mammalian bipolar cells is often based on three variables: axon terminal depth, axon arbor thickness, and axon arbor area (Cohen and Sterling, 1990; Boycott and Wässle, 1991; Euler and Wässle, 1995; Badea and Nathans, 2004; Ghosh et al., 2004; Li et al., 2004; Pignatelli and Strettoi, 2004). Previously, we successfully used these three variables plus the number of axonal ribbons for classifying mouse and macaque OFF bipolar cells (Tsukamoto and Omi, 2014) and macaque ON bipolar cells (Tsukamoto and Omi, 2016). Therefore, our standard procedure for clustering mouse ON bipolar cells used these four variables. However, the procedure was insufficient for type 5 and RB cells, so we added the vertical ribbon density profile for type 5 cells and the reciprocal synapse ratio for RB cells.

Using the side view of reconstructed bipolar cells, we analyzed axon terminal depth (or the IPL axon length), which was measured as the distance from the INL–IPL border to the axon terminal tip. In a particular case, however, for the convenience of comparison with the previous literature, we used the distance from the axon terminal tip to the ganglion cell layer (GCL) as the alternative variable (**Figure 5A**). The second variable analyzed was axon arbor thickness, which was the distance from the top of the arbor to the axon terminal tip. The top was defined as the point at which two or more processes branched out from the axon cylinder or the highest edge of the arbor. Using the top view of reconstructed bipolar axon terminal arbors, we measured axon arbor area, which was defined as π/4 × (the major diameter) × (the minor diameter), assuming that the circumference was an ellipse.

Individual synaptic contacts were localized on the coordinate axes of transparent sheets or in the computer graphics framework. Quantitative data were collected in spreadsheets for statistical assessment. We used Statistica 06J (Statsoft Japan, Tokyo, Japan) for cluster analysis (Ward's joining method). We used Image J (National Institutes of Health, Bethesda, MD, USA) for determining the densitometry of electron micrographs. Quantitative data are presented as the mean ± standard deviation and number of samples (*n*) unless otherwise indicated. The difference between two groups was assessed by an unpaired two-tailed Student's *t*-test, where the difference for which ^*^p < 0.05 or ^**^p < 0.01 was considered significant at each level of confidence.

## Results

### Classification and characterization by axon terminal measurements

#### Side view of all types of bipolar cells

**Figure 2:**
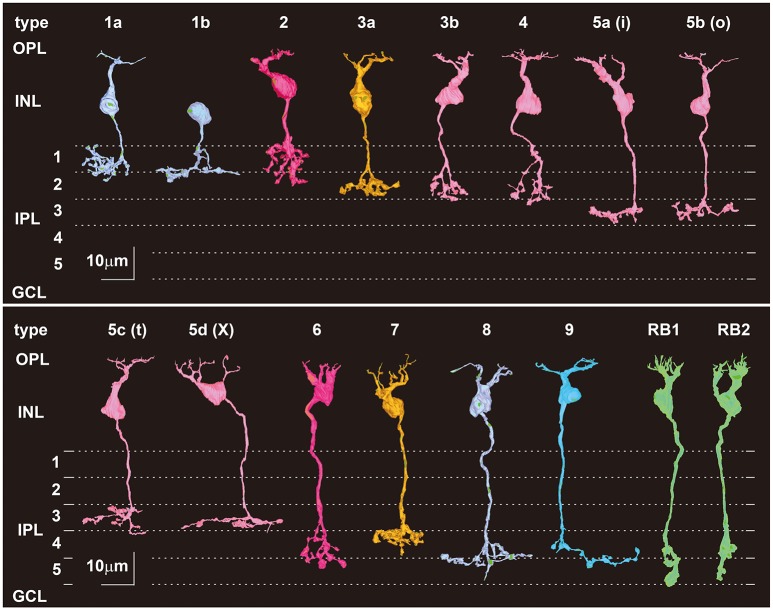
Morphology and stratification of all 15 types of mouse bipolar cells. The first six types (1a, 1b, 2, 3a, 3b, and 4b) are center-OFF response-type cells, which have axon terminals in the outer sublamina (strata 1 and 2) of the inner plexiform layer (IPL). The last nine types [5a, 5b, 5c, 5d, 6, 7, 8, 9, and rod bipolar (RB)] are center-ON response-type cells, which have axon terminals in the inner sublamina (strata 3, 4, and 5) of the IPL. Type 1b is morphologically unipolar but regarded as a bipolar cell class based on cell lineage. RB cells are divided into two groups: RB1, the cells of which have axon terminals extending upon or into the ganglion cell layer (GCL), and RB2, the cells of which have axon terminals beyond the GCL. The other 13 cell types (1a and 2–9) are cone bipolar cells. Each stratum of the IPL (1–5) is 8 μm thick.

Five types of OFF bipolar cells (1a, 2, 3a, 3b, and 4), one type of dendrite-less bipolar cell (1b), eight types of ON cone bipolar cells (5a, 5b, 5c, 5d, 6, 7, 8, 9), and two groups of RB cells (RB1 and RB2) are displayed in Figure 2. One aim of this study was to find similarities between ON and OFF cells and between mouse and monkey cells (Tsukamoto and Omi, 2014, 2015, 2016). For convenience, we present possible corresponding cells or cell groups in the same color. The classification of five types of OFF bipolar cells was performed in our previous study (Tsukamoto and Omi, 2014). For the present report, we reconstructed 19 ON cone bipolar cells, 18 RB cells, and 3 T1b cells from the same examination area as the previous study, in order to validate the classification of all bipolar cell types and to characterize cell type-specific synaptic connectivity. We adopted the terminology from Shekhar et al. (2016) by changing letters from uppercase to lowercase, such as 5A−5a. In addition, T5a, T5b, and T5c correspond respectively to 5i (inner), 5o (outer), and 5t (thick) types identified by Greene et al. (2016) and likewise T5d corresponds to X type identified by Helmstaedter et al. (2013).

Della Santina et al. (2016) identified a new type of neuron that they named a glutamatergic monopolar interneuron (GluMI). GluMI cells make glutamatergic ribbon synapses in the IPL. Electrophysiologically, this cell shows center-OFF responsiveness; morphologically, it has an axon but no dendrites. Using single-cell transcriptomics, Shekhar et al. (2016) revealed 15 types of bipolar cells, one of which has molecular markers of a bipolar cell but morphological characteristics of an amacrine cell. Because it has several pan-bipolar cell markers, the authors defined it as a type of bipolar cell and named it a BC1B cell. Based on its unique morphology, the BC1B cell is thought to correspond to the GluMI cell. In the same sampling area of the mouse retina we examined for the previous studies, we have now reconstructed three more cells similar to type GluMI or BC1B cells. Here, this novel type will be referred to as T1b, and cells previously defined as T1 will be referred to as T1a.

Although only one RB cell type is included on the list of bipolar cell types by Shekhar et al. (2016) and Pang et al. (2004) once described two distinct groups of RB cells: RB1 and RB2. Initially, we found two groups of RB cells. The axon terminals of RB cells in one group reached the GCL and had direct contact with somas of nearby ganglion or displaced amacrine cells. Those in the other group were slightly too short to reach the GCL and had no direct contact with any somas. Of 18 reconstructed RB cells, 11 cells belonged to RB1 and 7 cells to RB2.

The dendrites of ON and OFF cone bipolar and RB cells are located at the same level of the OPL, whereas their axons terminate type-dependently in different strata of the IPL. Axon terminals of OFF bipolar cells were located in strata 1 and 2. The mean stratification level of each type gradually deepened in rank order from T1a < T1b < T2 < T3a < T3b < T4. The axon terminals of T5a, T5b, T5c, and T5d cells commonly stratified in stratum 3. T7 cell axons terminated in stratum 4, whereas T6, T8, and T9 cell axons reached the upper half of stratum 5. The RB1 cell axon reaches the GCL and mostly crosses the IPL–GCL border, but the RB2 cell axon does not reach the IPL–GCL border. The mean values of their IPL axon lengths were significantly different (RB1: 41.2 ± 1.7 μm, *n* = 11; RB2: 38.0 ± 2.0 μm, *n* = 7; *t*-test: ^*^p = 0.002).

Axon arbor thickness of OFF bipolar cells ranged from 7 to 20 μm, and the mean value varied in rank order from T1a ~ T1b ~ T3a < T3b < T2 < T4. Axon arbor thickness of ON cone bipolar cells ranged from 5 to 19 μm, and the mean value varied in rank order from T5d < T5a < T7 ~ T5b ~ T5c < T9 < T8 < T6. Axon arbor thickness of RB1 cells ranged from 9 to 18 μm and from 10 to 14 μm for RB2 cells. There were no significant differences between the mean values (RB1: 13.6 ± 2.7 μm, *n* = 11; RB2: 12.3 ± 1.5 μm, *n* = 7; *t*-test: *p* = 0.2).

#### Distribution of axonal ribbons

**Figure 3:**
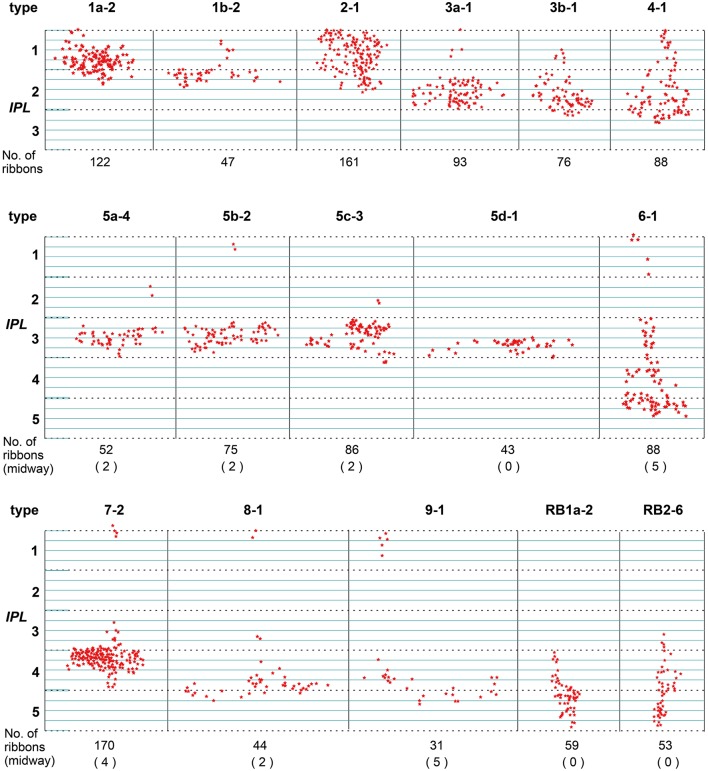
Side view of the axonal ribbons of each type of bipolar cell projected on the vertical plane. Stars each indicate the site of an axonal ribbon synapse with amacrine and/or ganglion cells. The number of axonal (midway + terminal) ribbons is written underneath each cell and the number of only midway ribbons is in each parenthesis. The axonal ribbons of OFF bipolar cells are confined to strata 1 and 2, and the outer half of stratum 3. The axonal ribbons of most ON bipolar cells are categorized into two, a major group of terminal ribbons in the ON sublamina and a minor group of midway ribbons in the OFF sublamina. The terminal ribbons are located largely in stratum 3 for types 5a−5d, strata 3 and 4 for type 7, and strata 3 and 4, and the outer half of stratum 5 for types 8–9. The midway ribbons are located largely in strata 1 or 2 for types 5a−5c and 6–9. The terminal ribbons are confined to the inner half of stratum 3, and strata 4 and 5 for RB1 and RB2. The vertical bin is 2 μm.

Synaptic ribbons in the axon terminal arbor of bipolar cells are shown in Figure 3 by projection onto a vertical plane with markers. The profile composed of all the ribbon markers characteristically resembles the terminal arbor shape for each cell type (Figure 2). The number of synaptic ribbons in the whole axon depended on cell type. The number of ribbons in OFF bipolar cells ranged from 47 to 177, and the mean value varied in rank order from T1b < T3a ~ T3b ~ T4 < T1a < T2. In ON cone bipolar cells, the number of synaptic ribbons ranged from 31 to 170, and the mean value varied in rank order from T9 < T5d ~ T8 < T5a < T5b < T5c ~ T6 < T7. In RB1 and RB2 cells, the number of synaptic ribbons ranged from 47 to 70 and from 52 to 60, respectively. There was no significant difference between the mean values (RB1: 54.4 ± 6.8 μm, *n* = 11; RB2: 55.3 ± 3.5 μm, *n* = 7; *t*-test: *p* = 0.7).

T6–T9 ON cone bipolar cells had axonal ribbons in the OFF sublamina as well as in the ON sublamina. Major groups of axonal ribbons were situated in IPL strata 3–5 and minor groups in stratum 1. T6, T7, T8, and T9 cells had 77 (mean, *n* = 3), 147 (mean, *n* = 3), 42 (*n* = 1), and 26 (*n* = 1) ribbons in the major groups, and 6, 3, 2, and 5 ribbons in the minor groups, respectively. A number of ribbon synapses in ON cone bipolar cells in the OFF sublamina of the IPL have also been reported in monkeys (Calkins et al., 1998) and rabbit retinas (Hoshi et al., 2009; Kim et al., 2012; Lauritzen et al., 2013).

#### Axon terminal territories of bipolar cells

**Figure 4:**
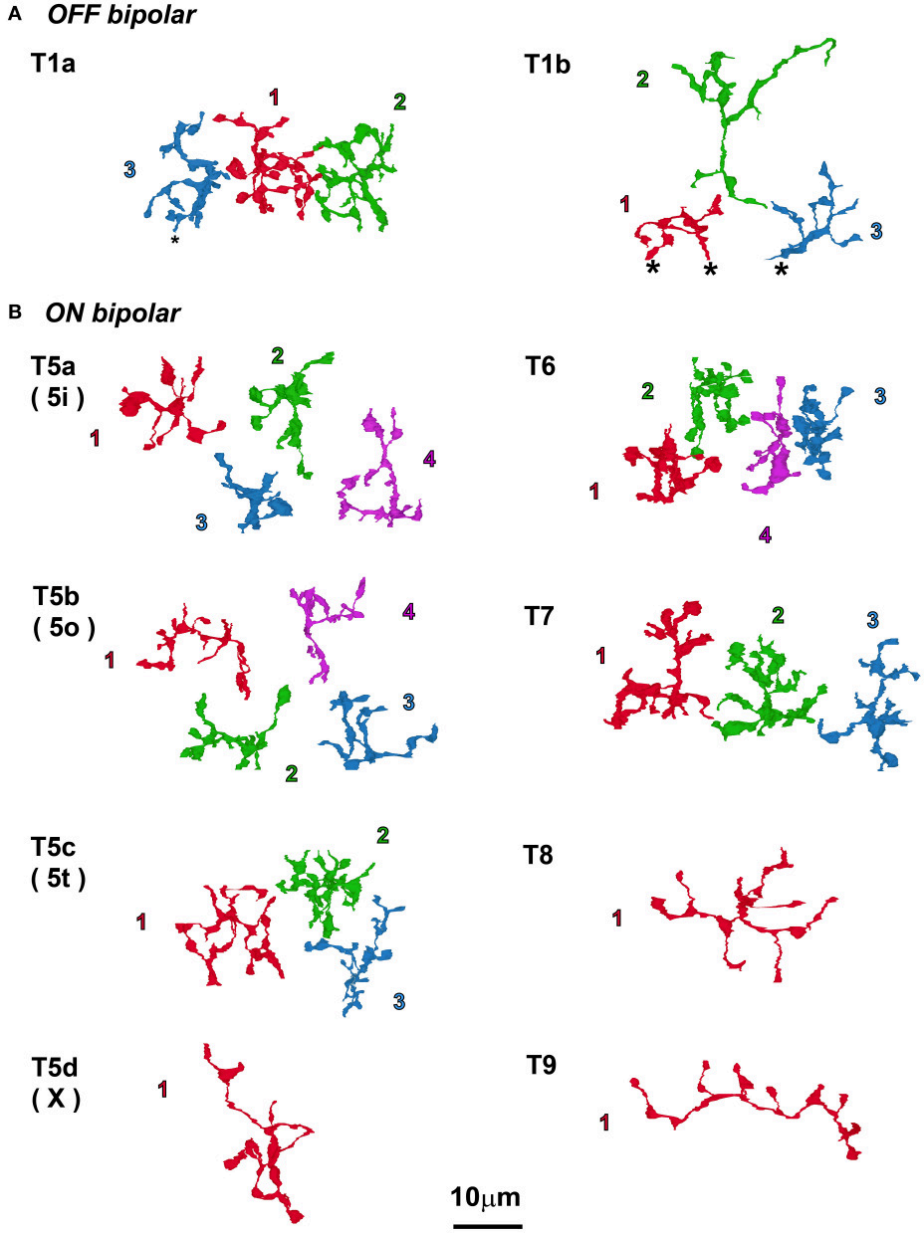
Top view of axon terminal arbors of bipolar cells that all coexist in the same examination area. **(A)** Only two types of OFF bipolar cells are shown here, so the novel type 1b cell observations are compared with the previously published type 1a cell description (Tsukamoto and Omi, 2014). **(B)** The eight types of ON bipolar cells; in this area, there was one cell each of types 5d, 8, and 9, and 3–4 cells each for all of the other types. Cells are displayed with the serial numbers used in other figures and colored for clarity. Asterisks (^*^) indicate the end of the series of electron micrographs.

Top-view profiles of axon terminal arbors of two types (T1a and T1b) of OFF bipolar cells and eight types (T5a–T9) of ON cone bipolar cells are displayed in Figure 4. Because those of five types of OFF cone bipolar cells (T1a, T2, T3a, T3b, and T4) have been previously described (Tsukamoto and Omi, 2014), we describe only the T1b type together with T1a for comparison. One T1b cell (Tb1-2) was completely reconstructed, but two other reconstructed cells (T1b-1 and T1b-3) were only partially included in the series. We sampled three to four cells each for types T5a, T5b, T5c, T6, and T7 but only one cell each for types T5d, T8, and T9 in the same area. The axon arbor area of the ON cone bipolar cells ranged from 90 to 420 μm^2^ and varied in rank order from T6 < T5a ~ T5b ~ T5c ~ T7 < T5d ~ T8 ~ T9. In each group of the same cell type, axon arbors were territorial; axonal arbors did not overlap with neighboring axonal arbors in the group.

#### Cluster analysis of ON cone bipolar cells using standard variables

**Figure 5:**
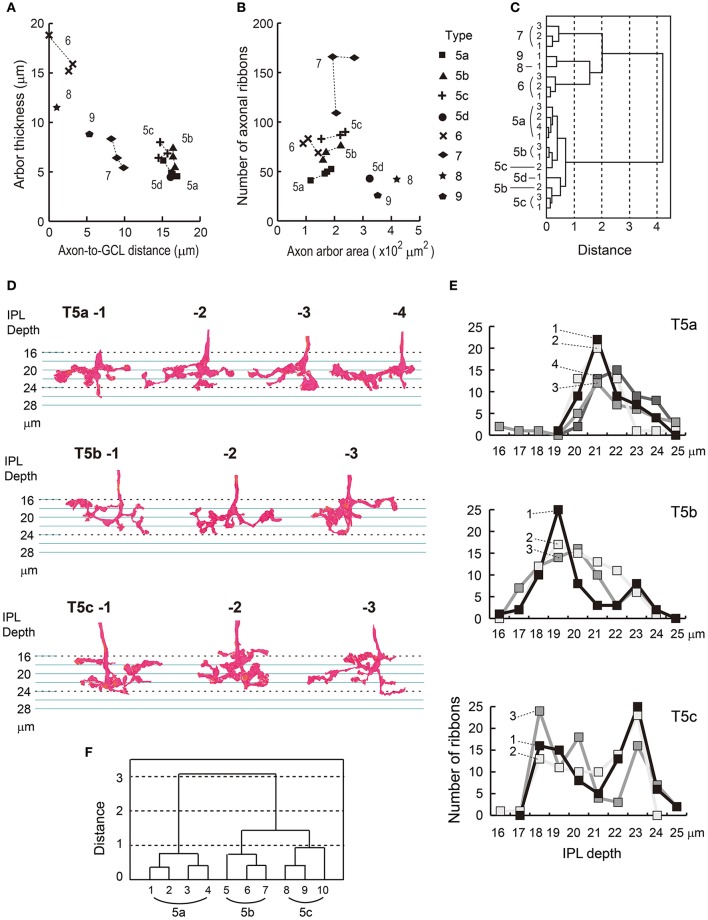
Cluster analysis of eight types of ON bipolar cells and a reexamination of type 5a, 5b, and 5c cells. **(A)** The scatter plot shows thickness of an axon arbor vs. the distance between the axonal tip and the ganglion cell layer. **(B)** The scatter plot shows the top-view area of an axon arbor vs. the number of synaptic ribbons. **(C)** A dendrogram of cluster analysis (Ward's method) of 19 ON bipolar cells using the four variables plotted in **(A**,**B)**. Two groups of cells (types 5b and 5c) are intermingled, but other cells are separated into six distinct clusters (types 5a, 5d, 6, 7, 8, and 9). **(D)** Side-view profiles of axon terminal arbors of type 5a, 5b, and 5c cells. **(E)** The vertical distribution of the number of synaptic ribbons per μm along the axon. **(F)** A dendrogram of cluster analysis (Ward's method) of 10 type 5 bipolar cells, using the distribution patterns of synaptic ribbons in **(E)**.

We confirmed the classification of eight types of ON cone bipolar cells based on four axon terminal variables: depth, thickness, area, and number of ribbons. As mentioned, these variables are sufficient for discriminating between cell types to a degree. The utility of each feature for discriminating between cells can be visualized in scatter plots (Figures 5A,B). Despite some variability, T5b and T5c points are always close to each other. Indeed, cluster analysis using these four variables revealed that T5b and T5c cells are intermingled, whereas the other six types are separated (Figure 5C).

#### Cluster analysis of type 5 bipolar cells with another variable

Top and side views of the gross morphology of reconstructed axon terminals of type 5 cells (Figures 2, 4, 5D) are similar to those presented by Greene et al. (2016). Among several morphological features used in the analysis by Greene et al. the most discriminative was the density profile of axon terminal branchlets along the IPL stratification depth (Figure 3C in Greene et al., 2016). Therefore, we obtained corresponding data on density profiles of axonal ribbons along the IPL stratification depth for our sample cells (Figure 5E). T5a (5i) cells had a single peak at a depth of 21 μm. T5b (5o) cells had a single peak at 19 μm, which as 2 μm further out than the T5a peak at 21 μm. T5c (5t) cells had two peaks, at 18 and 23 μm. These results are consistent with data from Greene et al. (2016). A cluster analysis using our data on the density profile of axonal ribbons yielded three distinct clusters (Figure 5F).

### Reexamination of rod bipolar cell types

#### Intermediate group RB1b between RB1a and RB2

**Figure 6:**
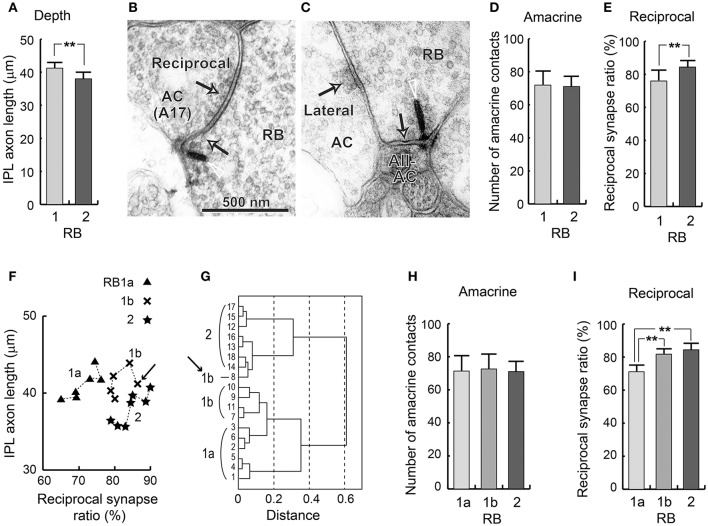
Groupings of RB cells. **(A)** The depth (IPL axon length) of RB cells as measured by the distance from the INL-IPL border to the axon terminal. **(B)** The electron micrograph of a ribbon synapse (arrowhead and arrow) from a RB cell axon terminal to an amacrine cell (possibly type A17), and a conventional synapse (arrow) reciprocally directed from an amacrine cell to the RB cell axon terminal. **(C)** The electron micrograph of a ribbon synapse (arrowhead and arrow) from the RB cell axon terminal to an AII amacrine cell (AII-AC), and a conventional synapse (arrow) laterally directed from another amacrine cell to the RB cell. **(D)** The total number of amacrine cell synapses directed to RB cells. **(E)** The percentage of ribbon synapses with reciprocal feedback from immediate amacrine cell dendrites. **(F)** A scatter plot of the two variables in **(D**,**E)** which shows significant differences among the three RB cell subtypes. **(G)** Clustering of the 18 RB cells using the two variables in **(F)**. **(H)** The total number of amacrine cell synapses directed to RB cells. **(I)** The percentage of ribbon synapses with reciprocal feedback from immediate amacrine cell dendrites. ^**^p < 0.01; unpaired two-tailed Student's *t*-test.

We measured the total axon length (from the axon hillock under the soma to the axon terminal tip) and IPL axon length (from the INL–IPL border to the axon terminal tip). There were no significant differences in total axon length between RB1 (57.7 ± 4.0 μm, *n* = 11) and RB2 cells (55.1 ± 1.8 μm, *n* = 7) (*t*-test, *p* = 0.083). However, the IPL axon lengths of RB cells were significantly greater (*t*-test, ^**^p = 0.003) for RB1 cells (41.2 ± 1.7 μm, *n* = 11) than for RB2 cells (38.0 ± 2.0 μm, *n* = 7), as shown in Figure 6A.

Pang et al. (2004) identified one more difference: RB1 cells have a higher threshold (0.044 Rh^*^/rod/s) before producing an inhibitory chloride current in response to light than RB2 cells (0.018 Rh^*^/rod/s). This raises the possibility that RB1 cells have fewer inhibitory (GABAergic and/or glycinergic) synapses than do RB2 cells. In the axon terminals of RB cells, there were mainly two types of synaptic contacts from amacrine cell processes: reciprocal synaptic contacts (Figure 6B), in which a few bilateral (ribbon and conventional) synapses exit, and lateral synaptic contacts (Figure 6C), in which no ribbon synapse exists in the neighborhood of a conventional synapse. Here we defined reciprocal synapses as ribbon synapses with reciprocal amacrine cell synaptic contacts at distances of <0.5 μm. Then we defined the reciprocal ratio as the ratio of the number of ribbons in the reciprocal synapse vs. the total number of ribbons. We also measured the total number of synaptic contacts between amacrine and RB cells, regardless of whether they were reciprocal or not. We initially assumed that these two measurements were counterparts to the threshold used by Pang et al. (2004). However, there was no significant difference in the number of amacrine cell synapses to RB cell axon terminals between RB1 cells (72 ± 8.7) and RB2 cells (71 ± 6.2; *t*-test, *p* = 0.8) (Figure 6D). By contrast, the reciprocal ratio was significantly higher in RB2 cells (84.4 ± 4.0, from 78 to 90%) than in RB1 cells (75.9 ± 6.6, from 65 to 87%; *t*-test, ^**^p = 0.008), although the overlapping range was not small (Figure 6E).

A scatter plot of IPL axon length vs. the reciprocal ratio shows that, for the reciprocal ratio, approximately half the RB1 cells were equivalent to RB2 cells, although, for IPL axon length, they were similar to the remaining half of the RB1 cells (Figure 6F). This remaining half of the RB1 cells were distinctly different from RB2 cells for both variables. Cluster analysis using these two variables divided 11 RB1 cells into two groups of 6 and 5 cells, which we defined as RB1a and RB1b, respectively (Figure 6G). The reciprocal ratio ranged from 65 to 76% for RB1a cells and from 78 to 87% for RB1b cells. Using this method, RB1b cells had a range of reciprocal ratios equivalent to that of RB2 cells. Notably, RB1b-8 (indicated by arrows in Figures 6F,G) appears to belong to the RB2 cell group. This discrepancy is due to the high reciprocal ratio (87%) of the RB1b-8 cell. Thus, based on this analysis, we categorized RB cells into three groups: RB1a, RB1b, and RB2. Amacrine cell synapses with RB cell axon terminals were equally abundant in these groups (Figure 6H). The ratio of reciprocal synapses of RB1b cells was significantly higher than of RB1a cells but equivalent to that of RB2 cells (Figure 6I). RB1a and RB1b cells had equivalent IPL axon lengths, as previously described. Thus, RB1b cells have hybrid characteristics of both RB1a and RB2 cells, indicating a continuity between RB1 and RB2 groups.

#### Survey of RB cells and synapse-like structures

**Figure 7:**
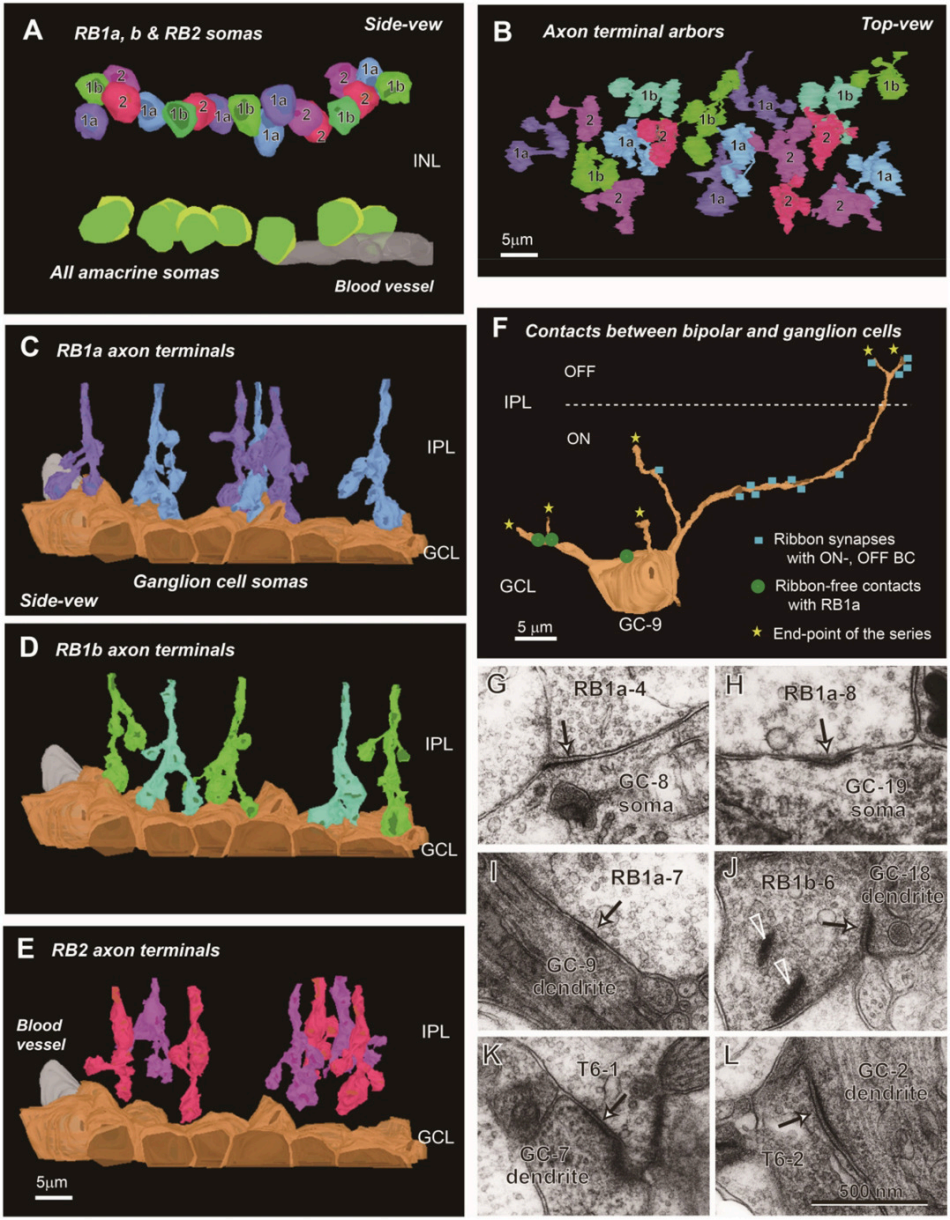
Spatial arrangement of RB1a, RB1b, and RB2 cells and fine structures of ribbon-free synapse-like contacts. **(A)** The somas of RB1a, RB1b, and RB2 cells are intermingled in the outermost sublayer of the INL. By contrast, the somas of AII amacrine cells are arrayed in the innermost sublayer of the INL. **(B)** The axon terminals of RB1a, RB1b, and RB2 cells are intermingled in the ON sublamina of the IPL. The somas (side view) and axon terminals (top view) of RB cells are not exactly in register because of the meandering courses of the axons. **(C–E)** The axon terminals of RB1a **(C)** and RB1b **(D)** cells extend downward and contact ganglion cell somas, whereas those of RB2 **(E)** cells are separate from ganglion cell somas. **(F)** The distribution of ribbon-associated synapses (blue squares) and ribbon-free synapse-like contacts (green discs) with a ganglion cell (GC-9: an ON-OFF type). The ganglion cell has ribbon-associated synapses with ON and OFF cone bipolar cells at the dendrites and ribbon-free synapse-like contacts with RB cells at the somas and dendrites. **(G–L)** Electron micrographs of ribbon-free synapse-like contacts (arrows) at bipolar cell axon terminals between RB cells and ganglion cell somas **(G,H)**, between RB cells and ganglion cell dendrites **(I,J)**, and between T6 cells and ganglion cell dendrites **(K,L)**. In **(J)** there are synaptic ribbons (arrowheads) nearby but separate from the ribbon-free synapse-like contact.

The spatial arrangement of the three groups of RB cells is shown in Figure 7. These 18 cells were contiguously arrayed, as shown in the two layers of the cell somas (Figure 7A) and axon terminal arbors (Figure 7B). Axon terminals of RB1a and RB1b cells reached the GCL and made contact with cell somas that are mostly ganglion cells but may be partly displaced amacrine cells. The axon terminals of RB2 cells did not reach the GCL. Consequently, they had no contact with cell somas (Figures 7C–E).

Next, we asked whether there were any synaptic structures in the contact area between RB1 cell axon terminals and ganglion cell somas (Figure 7F). Some sites did have postsynaptic density-like membrane densification (Figures 7G,H). In the cytoplasm on the presynaptic side, there were diffusely scattered synaptic vesicles but no clear aggregations of synaptic vesicles. Similar structures were found between RB cell axon terminals and ganglion cell dendrites (Figures 7I,J) and between T6 bipolar cell axon terminals and ganglion cell dendrites (Figures 7K,L). However, the frequency of occurrence of such ribbon-free synapse-like structures was very small in RB1a cells (1.6 ± 1.5 per axon, *n* = 6) compared with that of usual axonal ribbon synapses (56.8 ± 8.2 per axon, *n* = 6).

#### Cluster analysis of RB cells using standard variables

**Figure 8:**
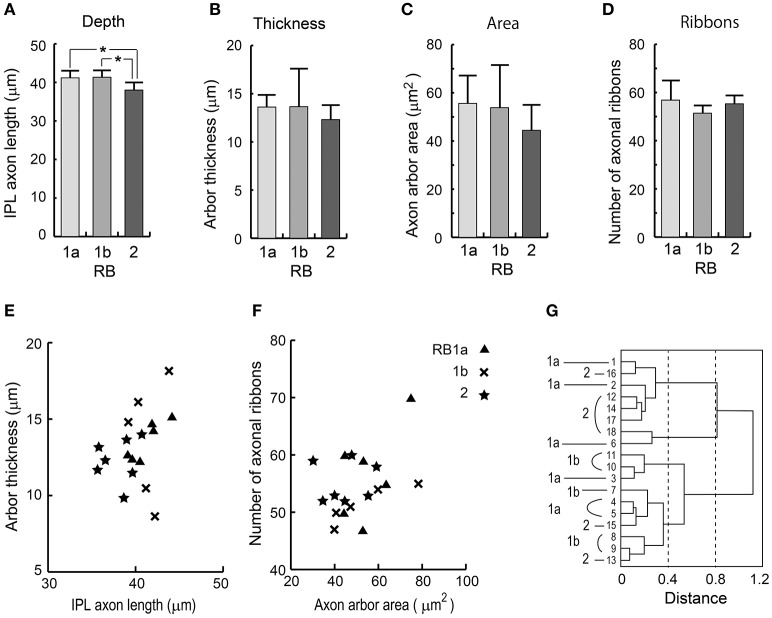
Quantitative assessment of RB1a, RB1b, and RB2 cells. **(A)** The depth as measured by the distance from the INL-IPL border to the axon terminal. **(B)** The vertical thickness of an axon terminal arbor. **(C)** The area of the top-view terminal arbor. **(D)** The number of synaptic ribbons in the axon. **(E)** A scatter plot of the two variables in **(A**,**B)**. **(F)** A scatter plot of the two variables in **(C**,**D)**. **(G)** Clustering of 18 RB cells using the four standard morphological variables in **(A–D)**. ^*^p < 0.05; unpaired two-tailed Student's *t*-test.

Based on axon terminal depth, the RB2 cell type was significantly different from the RB1a and RB1b cell types (Figure 8A; *t*-test, ^*^p = 0.014 for both RB1a–RB2 and RB1b–RB2). However, there were no significant differences in axon arbor thickness (Figure 8B), axon arbor area (Figure 8C), and the number of axonal ribbons (Figure 8D) (*t*-test, *p* > 0.05 for all pairs of the three groups). This is demonstrated in scatter plots showing combinations of two variables (Figures 8E,F). To determine whether RB cells should be classified into more than two types, we performed cluster analysis using these four variables. The analysis revealed no self-consistent separations (Figure 8G). All three optional groups (RB1a, RB1b, and RB2) are intermingled in the dendrogram. The inseparability of these three groups reflects all the *t*-test results for these four variables.

### Output ribbon synapses and input amacrine synapses

**Figure 9:**
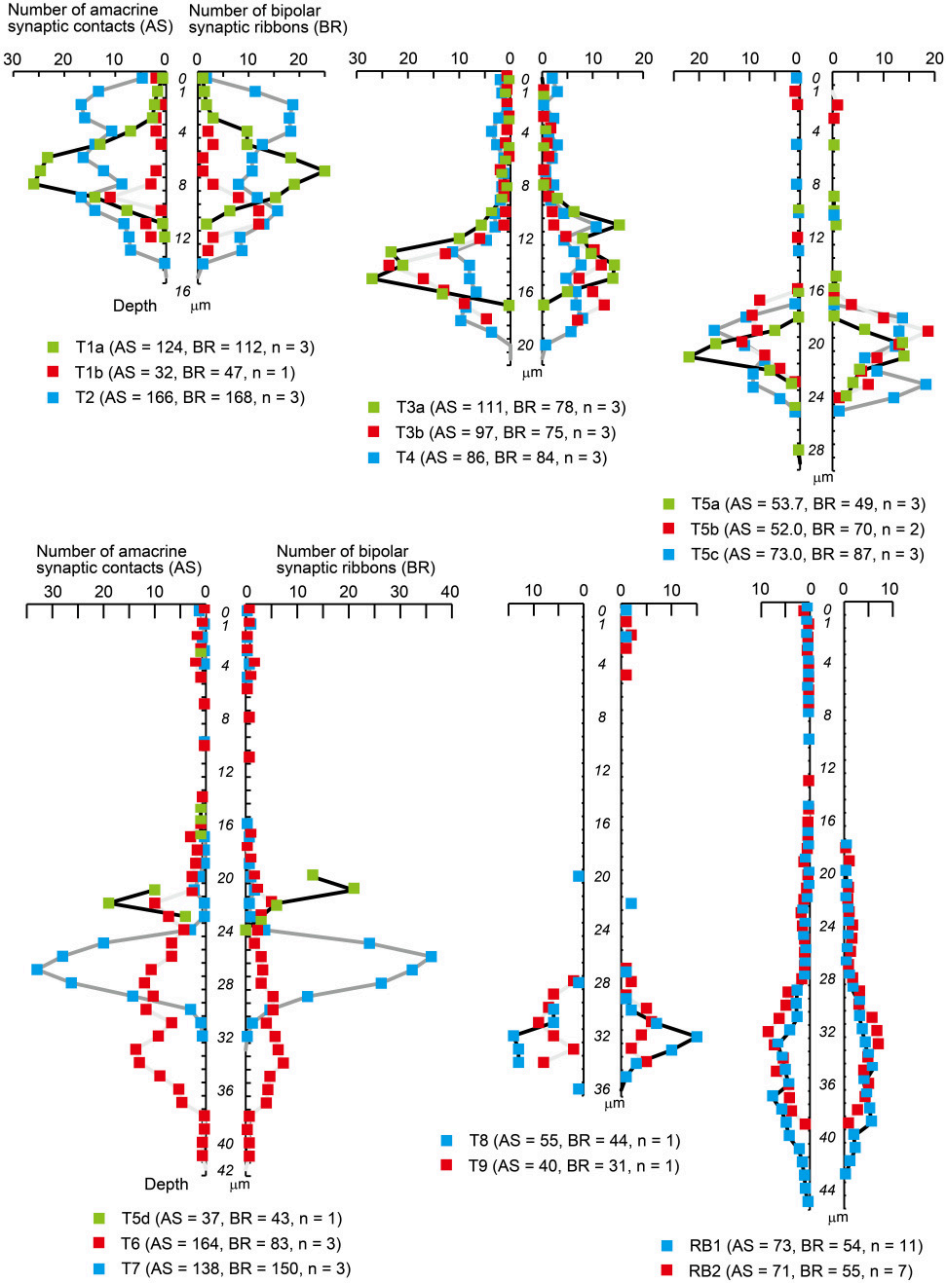
Density profiles of input amacrine cell synapses and output synaptic ribbons along the axon of each bipolar cell type. Only those points with positive values are designated with colored squares. The vertical axis shows the IPL depth with units of 1 μm; *0* indicates the extra bin above the INL–IPL border, *1* indicates the first bin from 0 to 1 μm, *4* indicates the fourth bin from 3 to 4 μm, and so on. The horizontal axis shows the mean number of amacrine synaptic contacts (AS, left) and bipolar axonal ribbons (BR, right) in each 1 μm bin. Numbers in underneath parentheses indicate the means of all amacrine synapses (AS) and all bipolar ribbons (BR) with the number of cells (n).

All types of bipolar cells have conventional synapses for input from amacrine cells and output ribbon synapses to ganglion and/or amacrine cells along their axons. We identified individual conventional amacrine cell synaptic contacts with bipolar cell axons throughout the IPL. Locations of individual ribbon contacts were also examined as previously described (Figure 3). The IPL depth of a contact point was defined as the distance from the INL–IPL border (0 μm) to that contact point. The maximal limit corresponds to the IPL–GCL border (40 μm). The density (number of contacts per μm) of input amacrine synaptic contacts and bipolar axonal ribbons are plotted on the left and right sides, respectively, along a common vertical axis in Figure 9. The contact density profile resembles the terminal arbor profile for both amacrine contacts and axonal ribbons. This may reflect the following: (i) synaptic contacts are almost evenly spaced on the surface of the axon terminal and (ii) the surface area of the axon terminal portion varies depth-dependently with the extent of arborization of that portion.

In the outer sublamina, each type of OFF bipolar cell has unique symmetrical density profiles of both amacrine contacts and axonal ribbons over the IPL axon length. Likewise, in the inner sublamina, each type of ON bipolar cell has unique symmetrical density profiles of both amacrine contacts and axonal ribbons. However, in the outer sublamina, ON bipolar cells show some irregular profiles. Five types (5a, 5b, 5c, 6, and 7) of ON bipolar cells have both amacrine contacts and axonal ribbons, two types (8 and 9) have only axonal ribbons, and two types (5d and RB) have only amacrine contacts along the axons passing through the outer sublamina.

**Figure 10:**
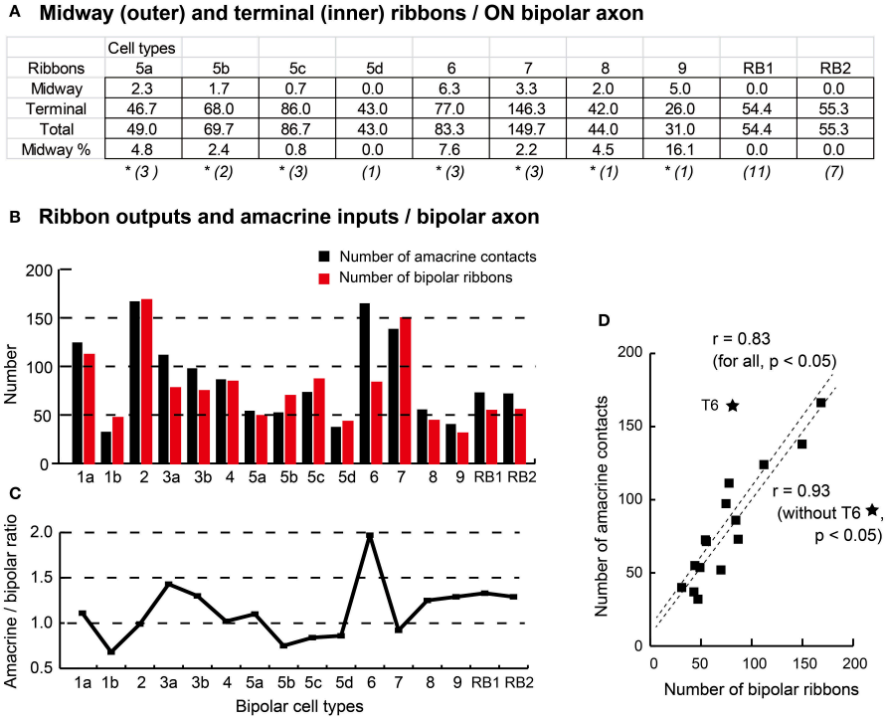
Proportion of midway and terminal ribbons **(A)** and correlation of amacrine input and bipolar output **(B–D)**. **(A)** Summary of the ON bipolar cell type-dependent distribution of the midway ribbons in the outer (OFF) sublamina and the terminal ribbons in the inner (ON) sublamina of the IPL. Percentage (%) is derived from the outer to total ratio. The number of cells is shown in each parenthesis, and an asterisk (^*^) means the existence of midway ribbons. **(B)** A histogram of the number of input amacrine cell synaptic contacts (black) and the number of output bipolar cell synaptic ribbons (red). **(C)** A line chart of the ratio of amacrine cell synapses to axonal ribbons across bipolar cell types. **(D)** A scatter plot with regression lines showing Pearson's correlation coefficient between the number of amacrine cell synapses and the number of bipolar cell ribbons.

Here, we call the ectopic ribbons located along the axon segment passing through the outer (OFF) sublamina “midway ribbons” and the entopic ribbons at the terminal arbor in the inner (ON) sublamina “terminal ribbons.” Seven types (5a, 5b, 5c, 6, 7, 8, and 9) of ON bipolar cells have midway ribbons but one type (5d) has no midway ribbon. In Figure 3, each of cells T5a-4, T5b-2, T5c-3, and T8-1 has two midway ribbons, cell T7-2 has four midway ribbons, and each of cells T6-1 and T9-1 has five midway ribbons. By adding more sample cells, their mean values are shown in Figure 10A. The mean percentage of midway ribbons to total ribbons per bipolar cell varies considerably, ranging from 0 to 16%.

Bipolar cell ribbon synapses release the excitatory neurotransmitter L-glutamate, whereas conventional amacrine cell synapses release the inhibitory neurotransmitters GABA or glycine. Therefore, the ratio of the number of amacrine cell contacts to the number of ribbons should be a useful morphological indicator of the balance between inhibitory and excitatory actions. Figure 10B shows a histogram of the number of amacrine cell contacts and the number of bipolar cell ribbons for all types of bipolar cells. These data are replotted as ratios in Figure 10C. These ratios are all around one: T1b, T5b, and T5c cells have ratios lower than one, T1a, T2, T4, T5a, and T7 cells have ratios close to one, and T3a, T3b, T8, T9, and RB cells have ratios between one and two. T6 cells have a ratio close to two. Both amacrine contact number and bipolar ribbon number are generally covariable. Indeed, Pearson's correlation coefficient for the variables of all bipolar cell types is 0.83. By excluding the outlying T6 case from the data set, the correlation coefficient for all the other cases increases to 0.93 (Figure 10D).

### Divergence of RB cell signals via AII amacrine cells

The signal derived from a single rod is dominantly directed to a few AII amacrine cells via two RB cells (Tsukamoto and Omi, 2013). AII amacrine cells are postsynaptic to RB cells via ribbon-associated contacts but not presynaptic to RB cells via any contacts. AII amacrine cell output involves the forward distribution of rod-driven signals to both OFF and ON pathways. AII amacrine cells have sign-inverting chemical synapses not only to OFF cone bipolar cells but also directly to OFF dendrites of ganglion cells. They also have gap junctions for sign-converting electrical coupling with ON cone bipolar cells (Figure 1).

**Figure 11:**
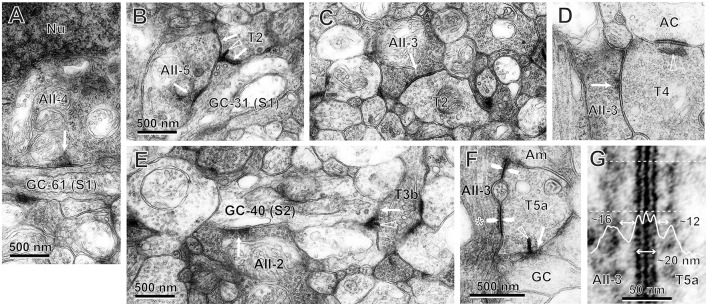
Electron micrographs of synaptic contacts (arrows) between bipolar cells of particular types (TNo.), AII amacrine cells (AII-cell number), and ganglion cells (GC-cell number) in stratum 1 (S1) or 2 (S2) of the IPL. Arrowhead: synaptic ribbon. **(A)** AII-4 has an output synapse to the dendrite of GC-61 in S1. **(B)** Both AII-5 and T2 cells have output synapses to the dendrites of GC-31 in S1. The T2 cell also has a ribbon synapse to the AII-5 cell. **(C)** The T2 cell has an input synapse from an AII amacrine cell. **(D)** The T4 cell has an input synapse from an AII amacrine cell, as well as a ribbon synapse to another amacrine cell. **(E)** Both AII-2 and T3b cells have output synapses to the dendrites of GC-40 in S2. **(F)** Gap junctions (pairs of arrows) between AII-3 and T5a cells. The T5a cell also has a ribbon synapse to a ganglion cell. **(G)** The magnified gap junction (asterisk of **F**) shows a striped pattern of three black lines with two intervening white lines. This gap junctional area is coated by fluffy subsurface material with spaces of different sizes on both sides in the cytoplasm. The density profile along the line perpendicular to the cell membranes is the average across the area between the two dotted lines.

**Figure 12:**
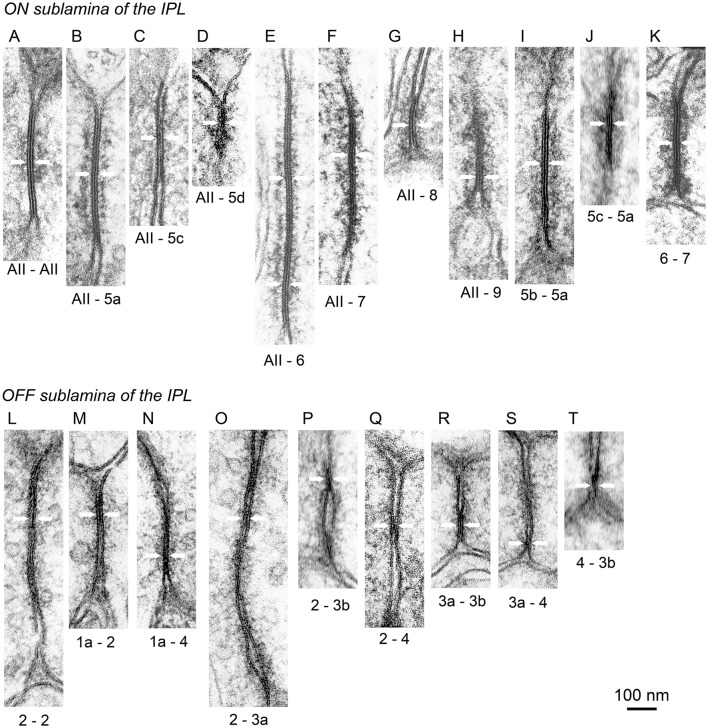
Electron micrographs of gap junctions (pairs of arrows) in the AII amacrine and bipolar cell system. **(A–K)** In the ON (inner) sublamina of the IPL, gap junctions were found between adjacent AII amacrine cells **(A)**, between AII amacrine and T5a **(B)**, T5c **(C)**, T5d **(D)**, T6 **(E)**, T7 **(F)**, T8 **(G)**, and T9 **(H)** cells, and between T5b–T5a **(I)**, T5c–T5a **(J)**, and T6–T7 **(K)** cells. **(L–T)** In the OFF (outer) sublamina of the IPL, gap junctions were found between adjacent T2 cells **(L)** and between T1a–T2 **(M)**, T1a–T4 **(N)**, T2–T3a **(O)**, T2–T3b **(P)**, T2–T4 **(Q)**, T3a–T3b **(R)**, T3a–T4 **(S)**, and T4–T3b **(T)** cells.

**Figure 13:**
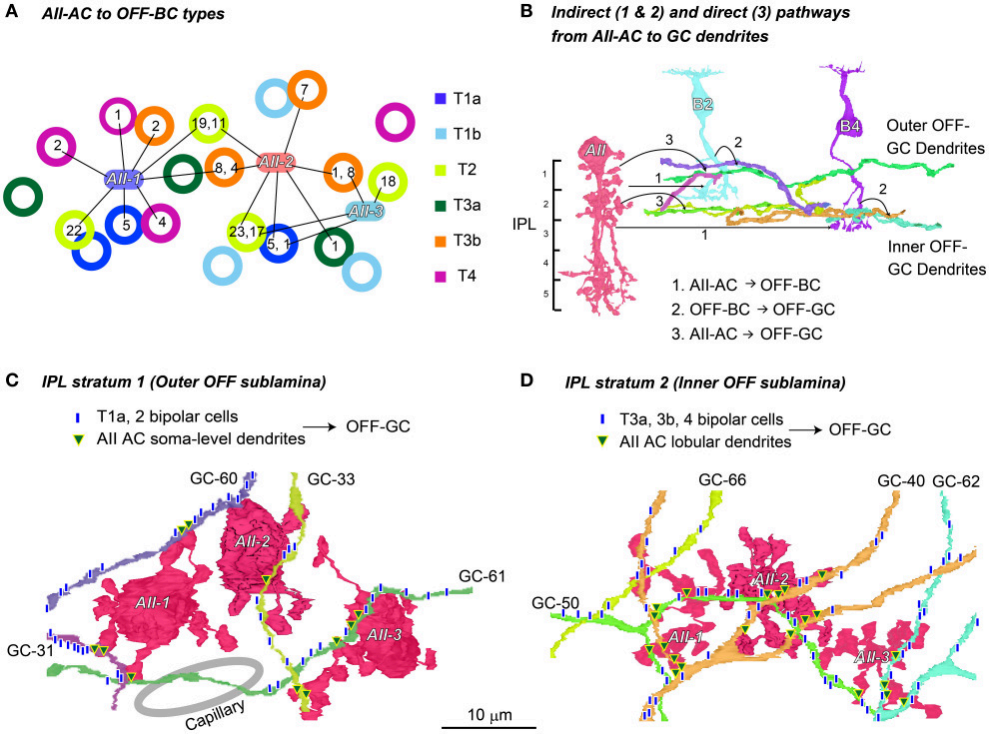
Two stratification levels for output to OFF ganglion cell dendrites from AII amacrine cells. **(A)** The number of synapses (shown in circles) from three AII amacrine cells (AII-1, −2, and −3) to different types of bipolar cells (labeled in different colors). **(B)** An illustration showing that both indirect (1 and 2: AII-AC → OFF-BC → OFF-GC) and direct (3: AII-AC → OFF-GC) pathways are implemented in either stratum 1 or stratum 2. **(C)** In stratum 1 of the IPL, dendrites of a group of ganglion cells contacted the soma-level dendrites of AII amacrine cells and the axon terminals of T1a and T2 cells. **(D)** In stratum 2 of the IPL, the dendrites of the other group of ganglion cells contacted the lobular dendrites of AII amacrine cells and the axon terminals of T3a, T3b, and T4 cells.

**Figure 14:**
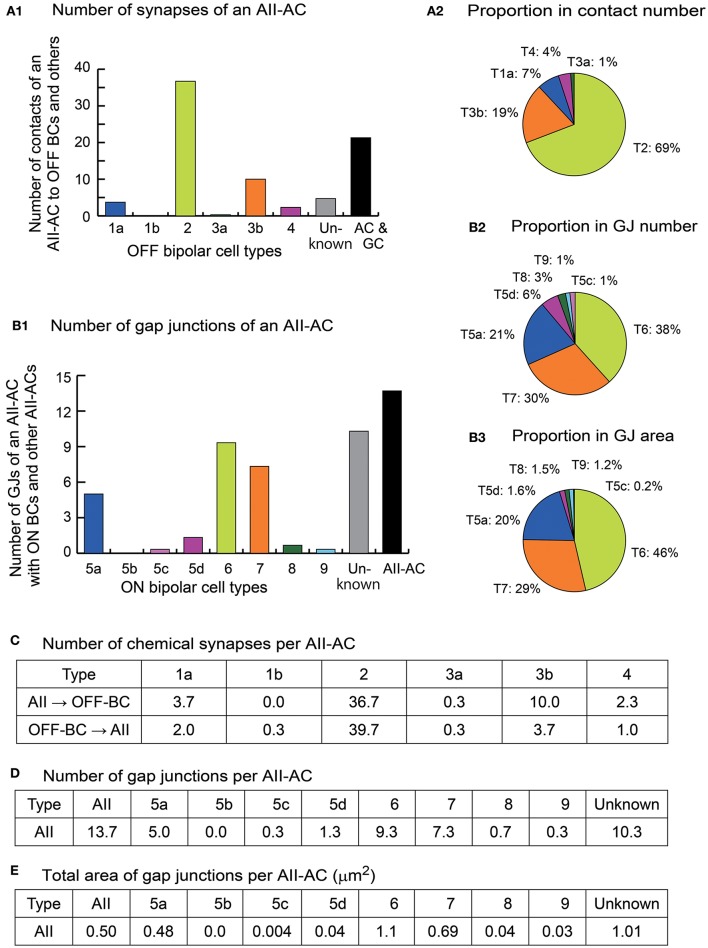
Type-dependent weighted outputs to bipolar cells from AII amacrine cells (AII-ACs). **(A-1)** The mean number of conventional synapses for output to different types of OFF bipolar cells, other amacrine cells, and ganglion cells per AII-AC (*n* = 3). **(A-2)** A pie chart showing the proportion of synapses to different types of bipolar cells. **(B-1)** The mean number of gap junctions with different types of bipolar cells and other AII-ACs per AII-AC (*n* = 3). **(B-2)** A pie chart showing the proportion of gap junctions with different types of bipolar cells. **(B-3)** A pie chart showing the proportion of gap-junction area with different types of bipolar cells. **(C)** The number of chemical synapses of AII-ACs and OFF bipolar cells per AII-AC. **(D)** The number of gap junctions of AII-ACs and ON bipolar cells per AII-AC. **(E)** The total area of gap junctions of AII-ACs and ON bipolar cells per AII-AC.

**Figure 15:**
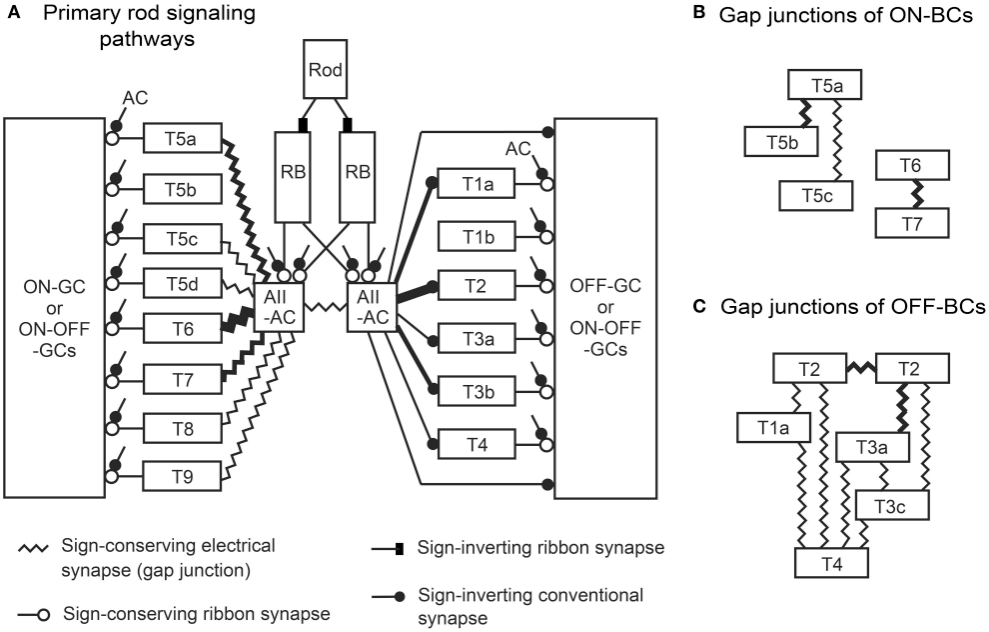
Model of the primary rod signaling pathways from a rod photoreceptor to ganglion cells via rod bipolar (RB) cells, AII amacrine cells, and all types of (cone) bipolar cells. **(A)** Each AII amacrine cell (AII-AC) had gap junctions with ON (cone) bipolar cells and conventional synapses with OFF (cone) bipolar cells, although only half are depicted. **(B)** Gap junctions between different types of ON bipolar cells (ON-BCs), which are connected to a network of AII-AC–ON bipolar cell gap junctions. **(C)** A network of gap junctions among OFF bipolar cells (OFF-BCs).

In the following section, we describe individual chemical synapses and gap junctions, based on the electron micrographs that we have obtained (Figures 11, 12). Next, we focus on the features of circuits in the OFF signal pathways (Figure 13). Finally, we assess the relative contributions of different types of bipolar cells to the OFF and ON pathways (Figures 14, 15).

#### Chemical synapses for output from AII amacrine cells

In stratum 1, the cell bodies of AII amacrine cells made somatodendritic synapses directed toward OFF dendrites of ganglion cells (AII-4 → GC-61 in Figure 11A), and the lobular dendrites of AII amacrine cells made dendrodendritic synapses directed toward OFF dendrites of ganglion cells (AII-5 → GC-31 in Figure 11B). The lobular dendrites of AII amacrine cells also made synapses directed toward type 1 and 2 OFF bipolar cells (AII-3 → T2 in Figure 11C). In turn, each of these bipolar cells formed ribbon synapses directed toward OFF dendrites of ganglion cells (T2 → GC-31 in Figure 11B). No synaptic contact was found between type 1b and AII amacrine cells.

In stratum 2 and the outer part of stratum 3, the lobular processes of AII amacrine cells made synapses directed toward type 3b and 4 OFF bipolar cells (AII-3 → T4 in Figure 11D). In turn, each of these bipolar cells formed ribbon synapses directed toward OFF dendrites of ganglion cells (T3b → GC-40 in Figure 11E). However, we only rarely observed AII amacrine cells with synapses directed toward type 3a OFF bipolar cells. In parallel in stratum 2, the slightly more vitreal lobular dendrites of AII amacrine cells made dendrodendritic synapses directed toward OFF dendrites of ganglion cells (AII-2 → GC-40 in Figure 11E).

#### Gap junctions for output from AII amacrine cells

##### Ultrastructure of gap junctions

An example of an ON bipolar cell (T5a) that receives signals from AII amacrine cells via gap junctions and sends signals to ganglion cells via ribbon synapses is shown in Figure 11F. In transmission electron micrographs, a gap junction between two adjacent cell membranes is visible as a hexalaminar structure of black–white–black–white–black lines. Notably, the outer layers of two apposed membranes are seen as a two-membrane-thick black line in center. Furthermore, there was some asymmetry in the cytoplasm between the two sides of the AII–bipolar cell gap junction. There was a layer of dense material at either side of the subsurface space of the junction, but the distance from the cytoplasm-side edge of the dense layer to the membrane surface appeared to be greater on the AII amacrine cell side than on the bipolar cell side, as shown in Figure 11G (~16 nm on the AII amacrine cell side vs. 12 nm on the bipolar cell side). Similar cytoplasmic asymmetry was also clearly found in AII amacrine–T6 cell gap junctions (Figure 12E). The same asymmetry between the two sides of AII amacrine–bipolar cell gap junctions was reported in cats (Kolb, 1979) and rabbits (Strettoi et al., 1992; Anderson et al., 2011).

By contrast, in case AII amacrine cells had gap junctions with adjacent AII amacrine cells (Figure 12A), no obvious asymmetry was observed in the cytoplasm between the two sides of the AII amacrine cell gap junction. Similar cytoplasmic symmetry was also observed in the T5b–T5a cell gap junction (Figure 12I). These cells have heterogeneity in molecular compositions: connexin 36 in AII amacrine cells vs. connexin 45 in most ON bipolar cells (Deans et al., 2002; Söhl et al., 2005; Dedek et al., 2006; Bloomfield and Völgyi, 2009). Recently, the expression of connexin 30.2 was observed in AII amacrine cells (Meyer et al., 2016). Because the AII amacrine cell junction has a higher permeability than does the AII amacrine–bipolar cell junction, these two pathways are thought to be differently regulated (Mills and Massey, 1995).

##### ON pathway

The distal dendrites of AII amacrine cells had gap junctions with T5a, T5c, T5d, T6, T7, T8, and T9 bipolar cells in strata 3 and 4 (Figures 12B–H) but no gap junction with T5b cells. Despite the lack of gap junctions with AII amacrine cells, T5b bipolar cells had gap junctions with T5a bipolar cells (Figure 12I). Gap junctions between different cell types were also found in T5a–T5c (Figure 12J) and T6–T7 (Figure 12K) cells. In contrast, no gap junctions were observed between ON bipolar cells of the same type. The absence of gap junctions between ON cells of the same type is consistent with the fact that axon terminals of the same type of ON bipolar cells are territorial and do not overlap, whereas axon terminals of different types of ON bipolar cells overlap in some places to enable direct contacts (Figure 4B).

##### OFF pathway

Gap junctions between OFF bipolar cells were more frequently observed than those between ON bipolar cells; the latter primarily had gap junctions with AII amacrine cells. Well-developed homocellular gap junctions were found between adjacent T2 cells (Figure 12L). Gap junctions between different cell types were observed in T1a–T2 (Figure 12M), T1a–T4 (Figure 12N), T2–T3a (Figure 12O), T2–T3b (Figure 12P), T2–T4 (Figure 12Q), T3a–T3b (Figure 12R), T3a–T4 (Figure 12S), and T4–T3b (Figure 12T) cells. These findings were substantiated by the array pattern of the axon terminals of these bipolar cells (Tsukamoto and Omi, 2014). Terminals of adjacent T2 cells occasionally contacted each other, whereas terminals of different types of OFF bipolar cells contacted each other more frequently.

#### Indirect and direct pathways from AII amacrine cells to OFF dendrites of ganglion cells at two stratification levels

Figure 13A shows the divergence of three AII amacrine cells (AII-1, -2, and -3) to OFF bipolar cells (types T1a, T1b, T2, T3a, T3b, and T4 in different colors) by their connections and number of synaptic contacts. For instance, AII-1 had the first and second largest number of outputs with T2 bipolar cells (22, 19), fewer outputs with T1a (5), T3b (8, 2), and T4 (4, 2) bipolar cells, but no contacts with T1b and T3a bipolar cells, thus a total of 55 contacts in these connections. Conversely, the T2 bipolar cell at the lower middle of Figure 13A received 23 contacts from AII-2 and 17 from AII-3. In general, the more inputs converged on a bipolar cell from the closer AII amacrine cells. AII amacrine cells made their first and second largest number of contacts (AII-1, 22, and 19; AII-2, 23, and 11; and AII-3, 18, and 17) to their preferred cell type, T2 bipolar cells.

AII amacrine cells indirectly relay rod-driven signals via OFF bipolar cell axon terminals to OFF dendrites of ganglion cells (Figure 13B, 1: AII amacrine cells → OFF bipolar cells and 2: OFF bipolar cells → OFF dendrites of ganglion cells). In addition, AII amacrine cells make direct synaptic contacts with OFF dendrites of ganglion cells (Figure 13B, 3: AII amacrine cells → OFF dendrites of ganglion cells).

Due to the limitation of our series, OFF dendrites of ganglion cells were not traced back to their somas. Nevertheless, these processes were identified as ganglion cells because of cytological features typical of ganglion cell dendrites, including numerous sites of postsynaptic densities, uniquely pale cytoplasm, and abundant microtubules. They also lacked presynaptic traits typical of amacrine cells. However, we could not determine whether the OFF dendrites originated in ON or ON–OFF ganglion cells. A number of such processes ran approximately horizontally in two different layers, roughly stratum 1 and stratum 2. The axon terminals of different OFF bipolar cell types also terminate in different levels (Figure 1).

In our examination area, we counted up to 25 ganglion cells that had their somas within the series of electron micrographs. In addition we found about 45 dendrites which were thought to belong to ganglion cells whose somas were not included in this series. ON bipolar cells mediated all the ON signals derived from the rod-RB-AII amacrine pathways via gap junctions to the ON dendrites of ganglion cells. OFF bipolar cells (except T1b) mediated most of the OFF signals derived from the rod-RB-AII amacrine pathways via chemical synapses to the OFF dendrites of ganglion cells. In parallel, part of the OFF signals was conveyed directly from AII amacrine cells to the OFF dendrites of some ganglion cells. Those ganglion cells appeared to utilize both indirect and direct connections with AII amacrine cells for the OFF signals. Here we picked up eight ganglion cells for the analysis of the indirect and direct pathways for the OFF signals in both strata 1 and 2.

In stratum 1 and the outer half of stratum 2 (0–30% of the IPL depth), four different ganglion cell dendrites (GC-31, -33, -60, and -61) received synaptic contacts from T1a and T2 OFF bipolar cells (Figure 13C), although contacts from T2 bipolar cells were much more dominant. These ganglion cell dendrites also received synaptic contacts from somas and dendrites of nearby AII amacrine cells. Collectively, 73 ribbon synapses from OFF bipolar cells and 39 conventional synapses from AII amacrine cells were made with dendrites of these four outer OFF ganglion cells in the same examination area. Thus, synapses from OFF bipolar cells outnumbered those from AII amacrine cells by 1.9-fold.

In stratum 2 and some marginal areas (20–45% of the IPL depth), four different ganglion cell dendrites (GC-40, -50, -62, and -66) received synaptic contacts from T2, T3a, T3b, and T4 OFF bipolar cells (Figure 13D) but few contacts from T2 cells. These ganglion cell dendrites also received input from nearby synapses with nearby lobular dendrites of AII amacrine cells. Collectively, 99 ribbon synapses from OFF bipolar cells and 49 conventional synapses from AII amacrine cells were made with the dendrites of these four inner OFF ganglion cells in the same sampling area. Thus, synapses from OFF bipolar cells outnumbered those from AII amacrine cells by 2.0-fold.

#### Divergence of AII amacrine cell outputs with different weights

Output from AII amacrine cells was largely divided into two pathways: the OFF pathway via conventional synapses and the ON pathway via gap junctions. Bipolar cell types contributing to each pathway were differently weighted. Their weights were assessed based on the average of three AII amacrine cells that were almost completely reconstructed as follows.

There were a total of 79 conventional synapses for output from an AII amacrine cell; 58 with OFF bipolar cell types (including five contacts with bipolar cells of unknown type) and 21 with amacrine or ganglion cells (Figures 14A-1, C). The percentages of synapses with each type of bipolar cell are shown in Figure 14A-2. The top three cell types carried 95% of signals from this AII amacrine cell; T2: 69%, T3b: 19%, and T1a: 7%. In contrast, T1b cells had no synapses.

Likewise, there were a total of 48 gap junctions with an AII amacrine cell; 34 with ON bipolar cell types (including 10 junctions with bipolar cells of unknown types) and 14 with neighboring AII amacrine cells (Figures 14B-1, D). The percentages of gap junctions with each type of bipolar cell are shown in Figure 14B-2. The top three cell types carried 89% of signals from this AII amacrine cell; T6: 38%, T7: 30%, and T5a: 21%. T5b cells had no gap junctions with AII amacrine cells.

However, in this context, the area of gap junctions may be more proportionately related to the conductance than the number of gap junctions. The sectional length of individual gap junctions varied considerably (Figures 12B–H). T6 cells had gap junctions with unusually long sectional lengths. In contrast T5d and T8 cells had gap junctions with very short lengths (Figures 12D, G). Therefore, we estimated the gap junction area by using the series of micrographs as a sum of the products of the sectional length times the sectional thickness. Depending on the cutting angles, approximately two-thirds of the gap junctions were measured. The percentages of gap junction area for each type of bipolar cell are shown in Figures 14B-3, E. Although the T5d cells receive 6% of the number of gap junctions, they receive only 1.6% of the area of gap junctions, a reduction of nearly 4-fold (Figures 14B-2, B-3). Based on gap-junction area, the top three cell types carried 95% of signals from this AII amacrine cell; T6: 46%, T7: 29%, and T5a: 20%.

## Discussion

### Summary of results

To describe parallel pathways involved in the divergence of rod-driven signals from an AII amacrine cell to bipolar cells (Figure 1), we first attempted to reconfirm the classification of 15 bipolar cell types. T5a, T5b, and T5c cells were distinctly clustered, whereas RB1 and RB2 were regarded to be in a continuum of variants of a single type. Incidentally ribbon-free synapse-like contacts were found between the axon terminals of RB and T6 cells and the somas and dendrites of ganglion cells. Next, we found a strong correlation between the number of amacrine cell inhibitory inputs and the number of bipolar cell excitatory outputs at axon terminals. Almost all ON cone bipolar cells were also found to have midway ribbons as well as terminal ribbons along their axons. Then, we assessed the connection strengths of AII amacrine cells to all types of cone bipolar cells for distributing rod-driven signals. The top three cell types in either the OFF (T2, T3b, and T1a) or the ON (T6, T7, and T5a) pathway have 95% of the total connection strength. Especially, T2 and T6 have the greatest relative strength (69 and 46%) among OFF and ON cone bipolar cell types respectively. In pathways from AII amacrine cells to OFF dendrites of a group of ganglion cells, the synapses for indirect transfer (via OFF cone bipolar cells) outnumbered the synapses for direct transfer by 2-fold.

### Dendrite-less T1b and multiple T5 bipolar cells

T1b cells had no dendrites but did have ribbon synapses at axon terminals. The axonal ribbons were similar to those of other bipolar cell types. Because T1b cells have no dendrites for an input channel, it is reasonable to assume that they may have gap junctions with other OFF bipolar cells as an alternative input channel. However, we did not find any gap junctions between T1b cells and other types of OFF bipolar cells. T1b cells are thought to be shaped by inhibitory input from amacrine cells (Della Santina et al., 2016). However, they had no synaptic contacts with AII amacrine cells. Therefore, they may not be involved in relaying rod-driven signals. This peculiar cell type awaits further characterization in future studies.

Taking advantage of a large number of reconstructed cells, Greene et al. (2016) classified type 5 cells into three distinct subtypes based on a detailed analysis of the stratification density profile and the coverage factor. These results are consistent with our study, which identified characteristic stratification patterns of axonal ribbons and territorial arrangements of axon arbors for each of the three cell types. In addition, we found that AII amacrine cells could be distinguished by T5b and T5c cells because they have gap junctions with T5c cells but not T5b cells. Thus, we think that the type 5 group consists of three subtypes, T5a (5i), T5b (5o), and T5c (5t). Furthermore, our current nomenclature includes T5d (X), for a total of four subtypes of group 5 bipolar cells with axon terminals at the same stratification level.

### RB cell type

#### RB cells as a single type

We initially defined three optional groups of RB cells: RB1a cells, which contact ganglion cell somas and have a moderate reciprocal synapse ratio, RB1b cells, which contact ganglion cell somas but have a high reciprocal synapse ratio, and RB2 cells, which reside apart from the ganglion cell somas and have a high reciprocal synapse ratio. Thus, RB1b cells have characteristics intermediate to RB1a and RB2 cells. Cluster analysis using our four standard variables (terminal depth, arbor thickness, arbor area, and ribbon number) resulted in the intermingling of RB1a, RB1b, and RB2 cells. Therefore, we regard these three groups of RB cells as three variants of a single type.

#### Reciprocal synapse ratio

Light-evoked responses in RB cells may provide glutamatergic sign-conserving transmissions to nearby amacrine cell processes. In return, amacrine cells releases an inhibitory neurotransmitter that induces a hyperpolarizing response in RB cells via chloride channels. The information content may differ depending on whether Ca ions are provided by local AMPA receptors (Kolb and Nelson, 1981; Chávez et al., 2006) or by voltage-dependent Ca channels evoked by depolarization from afar (Schubert et al., 2013). The former encode temporal characteristics and the latter encode spatial characteristics. Both schemes are thought to exist in RB cell axon terminals. The subtle difference between RB1a and RB1b cells may reflect a fine tuning of such spatiotemporal properties.

#### Ribbon-free synapse-like contacts

Although axon terminals of RB1 cells contacted ganglion cell somas, there were no ribbon synapses at the membrane-to-membrane contact areas. Instead, there were infrequent patches of dense, thick membrane on the ganglion cell side. There were scattered synaptic vesicles but no aggregation of synaptic vesicles in the cytoplasm on the bipolar cell side. Such ribbon-free synapse-like structures were found at the somas and dendrites of ganglion cells. Even T6 bipolar cells had similar structures.

This structure is similar to the ribbon-free active zone of (rod and cone mixed input and ON response-type) Mb1 bipolar cells in the goldfish retina (Midorikawa et al., 2007). Protein kinase C activation selectively increases the number of docked vesicles at ribbon-free sites, and slow vesicle fusion is induced by Ca currents that diffuse in from nearby ribbons. The ratio of the ribbon-free to ribbon-associated synapses was ~1.0 in the goldfish Mb1 cell. The corresponding value was ~0.03 in the mouse RB1a cell. The difference is over 30-fold. Currently, we have no physiological or pharmacological characterizations of ribbon-free synapse-like contacts in the mouse retina. The ribbon-free synapse-like contacts in the mouse RB cell terminal seem to be a rudimentary phylogenetic structure. Nevertheless, we cannot exclude the possibility that this mouse ribbon-free synapse-like structure has a synaptic function.

### Bipolar cell parallel processes with inhibitory regulation and electrical coupling

#### Diverse parallel processes

Field et al. (2009) showed that rod signals diverge to most or perhaps all ganglion cell types in the peripheral primate retina. Our previous study (Tsukamoto and Omi, 2013) showed that the signal from a single rod is represented as more than 100 replicates at the axon terminal ribbon synapses of two RB cells, which are directed mainly toward two or three AII amacrine cells. If almost all bipolar cell types have chemical or electrical synaptic contacts with AII amacrine cells, as observed in this study of the mouse retina, the diverse parallel processes of rod signals are logical. This analysis furthermore revealed the uneven contribution of those bipolar cell types to rod-driven AII amacrine cell signal processing (Figure 15). In addition, the OFF pathways consist of direct and indirect modes of signal transmission (Figure 13B). Ganglion cell dendrites in indirect pathways had twice as many synaptic contacts as direct pathways. In indirect pathways, signal transmission can be magnified with the aid of bipolar cells, but it takes more time. In direct pathways, transmission may be more prompt. Both modes act within this circuit (Figures 13C,D).

#### Strong correlation in the number of contacts between amacrine cell input and ribbon output

Franke et al. (2017) claimed that bipolar cell functional diversity is generated by the interplay of dendritic excitatory inputs and axonal inhibitory inputs. The characteristics of the dendrites produce type-specific responses including ON and OFF signs, fast and slow speeds, and narrow and wide fields. The dendritic excitatory inputs are thought to be conducted along the axon and represented by excitatory synaptic transmission at ribbon synapses. One of the global effects of amacrine inhibition at bipolar axon terminals was demonstrated as decorrelation of parallel pathways (Franke et al., 2017). Our data shows that both the number of synaptic ribbons and the number of amacrine contacts at the axon terminal of each bipolar cell type are almost equal (at most 2-fold difference) and mutually strongly correlated (Figures 9, 10). This correlation may imply that every axonal ribbon is readily accessible to a few nearby input contacts of amacrine cells which are depth-dependently chosen from among a vast list of amacrine cell types. That ribbon is also facing a few output targets which are likewise chosen from among many and various types of amacrine and ganglion cells. Under such circumstance, the formation of excitatory bipolar output at each synaptic site may be effectively regulated by a few nearby inhibitory inputs of amacrine cells. This hypothetical microcircuital scheme implies a two-step selection by type-specificity and site-specificity for conveying signals to amacrine and ganglion cells.

In general, glycinergic amacrine cells are the narrow-field type (Pourcho and Goebel, 1985; Menger et al., 1998) and GABAergic amacrine cells are the wide-field type (MacNeil and Masland, 1998). According to Ivanova et al. (2006), the application of GABA elicited inward currents from all 10 types of bipolar cells known at that time. However, the OFF and ON systems had different responses to glycine. OFF bipolar cells exhibited prominent glycine-induced currents, whereas ON bipolar cells exhibited very small glycinergic currents. The prominence of glycinergic responses in OFF bipolar cells is consistent with the abundance of synapses between AII amacrine cells and OFF bipolar cells (Figures 13A, 14A,C), because AII amacrine cells are characterized by the narrow-field dendrites having glycinergic output synapses.

#### Accessory ON signals by midway ribbon synapses in the OFF sublamina

The variety of the axonal ribbon outputs of ON bipolar cells is further enhanced by the midway ribbons that provide accessory ON signals in the outer (OFF) sublamina of the IPL. While midway ribbons were found in seven types (5a, 5b, 5c, 6, 7, 8, and 9) of ON bipolar cells (Figure 3, 10A), T6 cells had the largest number of midway ribbons per bipolar cell (mean: ~6) and a T9 cell recorded the highest percentage of midway to total ribbons (~16%). The midway ribbons in type 6 and 9 cells were previously recognized by other authors. Dumitrescu et al. (2009) observed midway ribbon synapses in the mouse retina and stated that those cells morphologically resembled type 6 cone bipolar cells but did not exclude their belonging to type 7 or 8. The authors showed their contacts with M1 melanopsin-expressing ganglion cells and dopaminergic amacrine cells and further confirmed that they were functionally ON bipolar cells because of their depolarizing light responses. Calkins et al. (1998) observed 3–7 midway ribbons per S-cone bipolar cell (T9 counterpart) in the macaque retina but did not identify the postsynaptic cell types.

The postsynaptic cell types of midway ribbon synapses were moreover identified in the rabbit retina. Hoshi et al. (2009) clarified that calbindin-positive ON cone bipolar cells made ribbon synapses in the OFF sublamina with bistratified diving ganglion cells in addition to the afore-mentioned two cell types. Rabbit retinal connectomics by Lauritzen et al. (2013) clarified several interesting findings. First, the authors claimed the existence of 15 types of rabbit bipolar cells (6 OFF, 8 ON, and 1 RB) with their own revision (addition of two novel types: CBb5w and CBb6) of the classification by MacNeil et al. (2004). The taxonomy of rabbit bipolar cells seems to be equivalent to that of mouse bipolar cells. Second, they had a collection of incidences showing that all eight types of ON cone bipolar cell make midway ribbons. Third, 36% of ON bipolar cells (51 of 141 cells) had one or more midway ribbons. It means that not all ON cone bipolar cells in a given type form midway ribbon synapses. Forth, RB cells do not provide particular accessory ON signals in the OFF sublamina, being consistent with our data. Fifth, they extended a list of the postsynaptic cell types to c-aminobutyrate (GABA)-positive amacrine cells, glycinepositive amacrine cells, other ON–OFF, and OFF ganglion cells.

In our analysis, all three T6 cells (T6-1, -2, and -3) were positive for the presence of midway ribbon (5, 5, and 9 ribbons) but only two of three T5c cells (T5-1, -2, and -3) were positive (0, 1, and 1 ribbon). Therefore, although cell T5d-1 (*n* = 1) had no midway ribbons, this does not exclude the possibility of other T5d cells having midway ribbons. In light of other studies, all ON cone bipolar cells may have the potential to make midway ribbon synapses to convey accessory ON signals in the OFF sublamina.

#### ON and OFF signal transmission through gap junctions

Mouse AII amacrine cells transmit rod-driven signals through gap junctions to seven types of ON bipolar cells (Figure 15A). The proportions of these pathways for electrical transmission appear to be uneven in both number and junctional area among different cell types. The area of gap junctions in AII amacrine cells are greatest with T6 bipolar cells (~46%, Figure 14B-3), moderate with types 7 (29%) and 5a (20%), smallest with types 5c, 5d, 8, and 9 (1–2%), and non-existent with type 5b. No gap junctions were found between AII amacrine and one-fifth of the population of ON bipolar cells (Petrides and Trexler, 2008). Disproportionate distributions of gap junctional pathways were also observed in the cat retina (Cohen and Sterling, 1990), where the number and size gap junctions in AII amacrine cells are greatest with type b1, moderate with b2 and b4, and non-existent with b3 bipolar cells. Cat type b1, b2, b3, and b4 bipolar cells appear to be similar to mouse T6, T8, T5b, and T5a bipolar cells, respectively, based on comparisons of stratification and arborization of their axon terminals and dendrites. Remarkably, type b3 bipolar cells have gap junctions with type b4 bipolar cells in the cat. Similarly, T5b cells have gap junctions with T5a cells in the mouse (Figures 12H, 15B). These findings suggest that T5b cells may communicate with AII amacrine cells through T5a cells (AII → 5a → 5b) in the mouse, as suggested for cat b3 and b4 cells (AII → b4 → b3; Cohen and Sterling, 1990).

The nine combinations of OFF bipolar cell types for gap-junction coupling are displayed in Figure 12L–T. A T2 cell may electrically couple its signal with neighboring T1a, T2, T3a, T3b, and T4 cells via gap junctions. Neighboring OFF bipolar cells also share their signals with even more cells via gap junctions (Figure 15C). Likewise, a T6 cell has gap junctions with AII amacrine cells. Because the AII amacrine cells have gap junctions with most of the ON bipolar cell types (Figures 12B–H, 15A) and also there are some bipolar–bipolar gap junctions (Figures 12I–K, 15B), the T6 cell may communicate with almost all neighboring ON bipolar cells via gap junctions. Thus, T2 and T6 cells are centrally located for sharing signals in each bipolar cell network of electrical couplings. Kuo et al. (2016) showed that electrical and chemical synapses work in concert to regulate glutamate release from ON cone bipolar cells.

#### Possible T7 cell pathway

Pan et al. (2016) demonstrated four intensity-response functions of mouse retinal ganglion cells with different threshold sensitivities: high sensitivity (HS), intermediate sensitivity (IS), low-intermediate sensitivity (LIS), and low sensitivity (LS). The first three categories reflect three different rod (scotopic) signaling pathways, and the last category reflects the cone (photopic) signaling pathway. By blocking inhibitory synapses, the authors examined how the masking inhibition by amacrine cells onto bipolar cell axon terminals was involved in controlling the sensitivity of different cohorts of ganglion cells. Dark-adapted alpha-ON ganglion cells showed threshold sensitivities consistently in the LIS range (Pan et al., 2016). Several studies imply the possible involvement of T7 cells in the tertiary ON rod pathway to alpha-ON ganglion cells.

Synaptic contacts between T7 cells and rods were first observed by SSTEM of wild-type and mGluR6-knockout mice and by fluorescence imaging of GUS-GFP mice (Tsukamoto et al., 2007). T7 cells project their dendrites mainly toward cone pedicles and, from there, a few tiny branches extend to the scleral side toward nearby rod spherules. Therefore, contact sites with rod spherules are frequently hidden behind cone pedicles in top-view light microscopy. Nevertheless, dendritic extensions of T7 cells that might be associated with rod spherules were repeatedly observed in the retinas of GUS-GFP adult mice (Keeley and Reese, 2010). Lee et al. (2011) also pointed out that a mature T7 cell exhibited a few contacts free of pedicles (i.e., with rod spherules) but whether immature T7 cells contacted rod spherules was unclear. According to our data (Tsukamoto and Omi, 2013), ~50 rod spherules have invaginating synapses with RB cells at 99 sites compared with T7 cells at 1 site. This factor may become the cause of the 2-log unit difference in the gain of photon absorption signals. This property is in line with the LIS range (Pan et al., 2016).

Pang et al. (2010) showed, using wild-type and connexin 36-knockout mice, that a subpopulation of ON cone bipolar cells (DBC_C1_) elicited responses in the scotopic light-intensity range, implying that they receive direct synaptic input from rods. The morphology of DBC_C1_ cells was similar to those of T6 and T7 cells. As shown in the present study, T6 and T7 cells are thought to be mutually communicable via gap junctions (Figure 12J). In wild-type mice, Pang et al. (2003) found that alpha-ON ganglion cells had mixed input from cones and rods. Rod input was once thought to be via the secondary pathway of cone–rod cell gap junctions (Cx36). However, recently, in connexin 36-knockout mice, Pan et al. (2016) found that alpha-ON ganglion cells still had scotopic sensitivity. The rod input in this case may be from tertiary pathways of T7 cells; one possible pathway is the connection of rod–(chemical)–T7–(chemical)–ganglion cell and the other possible pathway is the connection of rod–(chemical)—T7–(electrical)–T6–(chemical)–ganglion cell. Schwartz et al. (2012) demonstrated that the alpha-ON ganglion cell had a major input from T6 cells but also a minor input from T7 cells (<5% of the excitatory synapses). Concerning T6-T7 gap junctions, Han and Massey (2005) reported that T7 cells had connexin 36 instead of connexin 45 but were always colocalized with AII amacrine cells. Their finding implies that T7 cells may utilize connexin molecules different from Cx36 for coupling with T6 cells. The connection between T7 and ganglion cells remains to be fully elucidated (Lin and Masland, 2005).

### Major players in relaying rod-driven signals from AII amacrine cells

#### Comparison with Helmstaedter et al.'s data

The top three ON bipolar cell types, T6 (46%), T7 (29%), and T5a (20%), shared 95% of the total area of gap junctions of all ON bipolar cells with AII amacrine cells according to our data. This is consistent with data from Helmstaedter et al. (2013), who reported that T6 (2%) and T7 (1.4%) are the top two types. They used the percentage of total neuronal contact area of an AII amacrine cell as an indicator of the potential abundance of synapses. For comparison with data from Helmstaedter et al. for OFF bipolar cell types, some revision is necessary. Their type 1 cells (in their Figure 1C) should be type 2 and *vice versa* according to the original definition by Ghosh et al. (2004), who used the stratification level of the axon terminals as the major criterion for classification. In fact, the axon terminal of the type 2 cell was shown to be deeper than that of the type 1 cell (Ghosh et al., 2004). The classification using this Ghosh et al.'s criterion has been used by many other authors (Ivanova et al., 2006; Kim et al., 2014; Behrens et al., 2016).

After this revision, the data by Helmstaedter et al. (2013) indicate that the percentage of contact area varies in rank order from T2 (6.4%) > T1a (4.3%) > T3b (3.2%) > T4 (2.9%) > T3a (1.8%). From our data, the percentage of the total number of synapses between an AII amacrine cell and each type of bipolar cell varies in rank order from T2 (69%) > T3b (10%) > T1a (7%) > T4 (4%) > T3a (1%). The top three cell types, T2, T1a, and T3b, are the same in both data sets. T2 is first in both studies, although the second and third positions are switched. From our data, T3a cells have the smallest percentage (1%; except 0% with T1b cells). From their data, T3a cells also have the lowest value (1.8%). As a whole, measurements from these two studies are consistent.

#### Comparison of mouse T2 and T6 cells with macaque midget bipolar cells

Recoverin, an immunological marker, labels T2 bipolar cells in the mouse retina and flat midget bipolar (FMB) cells in the macaque retina (Ghosh et al., 2004). The convergence of cones to a bipolar cell is the smallest among OFF type cells for both T6 and FMB cells (Wässle et al., 2009). In the top view of the axon arbors, both mouse T2 cells and macaque FMB cells have similar varicosity profiles, which are characterized by a wide central area that is in conjunction with a relatively thick axon (Tsukamoto and Omi, 2014). T2 bipolar cells may correspond to FMB cells. Likewise, mouse T6 bipolar cells have a relatively thick axon, the smallest arbor area among ON type cells, and the largest cell density (Ghosh et al., 2004; Helmstaedter et al., 2013; Greene et al., 2016) as macaque invaginating midget bipolar (IMB) cells do (Tsukamoto and Omi, 2016). T6 cells are thought to correspond to macaque IMB cells.

Data from this study suggest that T2 and T6 bipolar cells work mainly in the scotopic condition. However, the macaque FMB and IMB cells work mainly in the photopic condition. This implies that T2 and T6 bipolar cells may also work mainly in the photopic condition and that FMB and IMB cells may also work mainly in the scotopic condition. Indeed, our preliminary observations of the macaque retina show that FMB cells have the most numerous conventional synapses with AII amacrine cells among OFF types, while IMB cells have the most numerous gap junctions with AII amacrine cells among ON types (unpublished data). These findings imply that the macaque FMB and IMB cells or the mouse T2 and T6 cells may work in either scotopic or photopic conditions, which alternate in a daily cycle. It seems highly likely that the most sensitive scotopic signal is conveyed to the center by ganglion cells that have the most numerous synaptic contacts with T2 and T6 cells (Neumann et al., 2016). This architecture is thought to be an example of the time-sharing work systems that allow the economical utilization of the limited resource of neural circuits.

## Author contributions

The authors had full access to all the data in the study and take full responsibility for the integrity and the accuracy of the data analysis. YT designed this study, took micrographs, acquired data, interpreted results, and wrote the manuscript. NO took micrographs, acquired data, and checked the manuscript.

### Conflict of interest statement

The authors declare that the research was conducted in the absence of any commercial or financial relationships that could be construed as a potential conflict of interest.
